# Dysfunction of the WT1-*MEG3* signaling promotes AML leukemogenesis via p53-dependent and -independent pathways

**DOI:** 10.1038/leu.2017.116

**Published:** 2017-05-02

**Authors:** Y Lyu, J Lou, Y Yang, J Feng, Y Hao, S Huang, L Yin, J Xu, D Huang, B Ma, D Zou, Y Wang, Y Zhang, B Zhang, P Chen, K Yu, E W-F Lam, X Wang, Q Liu, J Yan, B Jin

**Affiliations:** 1 Department of Hematology, the Second Affiliated Hospital, Institute of Cancer Stem Cell, Cancer Center, Dalian Medical University, Dalian, China; 2 Department of Hematology, the Second Affiliated Hospital, Institute of Hematopoeitic Stem Cell Transplantation of Dalian Medical University, Liaoning Hematopoeitic Stem Cell Transplantation Medical Center, Dalian Key Laboratory of Hematology, Dalian Medical University, Dalian, China; 3 Department of Neurosurgery, the Second Affiliated Hospital of Dalian Medical University, Dalian, China; 4 Department of Obstetrics and Gynecology, the Second Xiangya Hospital, Central South University, Changsha, China; 5 Department of Cellular Biology and Anatomy, Augusta University, Augusta, GA, USA; 6 Department of Surgery and Cancer, Imperial College London, London, UK

## Abstract

Long non-coding RNAs (lncRNAs) play a pivotal role in tumorigenesis, exemplified by the recent finding that lncRNA maternally expressed gene 3 (*MEG3*) inhibits tumor growth in a p53-dependent manner. Acute myeloid leukemia (AML) is the most common malignant myeloid disorder in adults, and *TP53* mutations or loss are frequently detected in patients with therapy-related AML or AML with complex karyotype. Here, we reveal that *MEG3* is significantly downregulated in AML and suppresses leukemogenesis not only in a p53-dependent, but also a p53-independent manner. In addition, *MEG3* is proven to be transcriptionally activated by Wilms’ tumor 1 (WT1), dysregulation of which by epigenetic silencing or mutations is causally involved in AML. Therefore *MEG3* is identified as a novel target of the WT1 molecule. Ten–eleven translocation-2 (TET2) mutations frequently occur in AML and significantly promote leukemogenesis of this disorder. In our study, TET2, acting as a cofactor of WT1, increases *MEG3* expression. Taken together, our work demonstrates that TET2 dysregulated WT1-*MEG3* axis significantly promotes AML leukemogenesis, paving a new avenue for diagnosis and treatment of AML patients.

## Introduction

Acute myeloid leukemia (AML) is the most common malignant myeloid disorder in adults, which is a heterogeneous clonal disorder of hematopoietic progenitor cells, with diverse biological, phenotypic and prognostic behaviors, and strikingly different outcomes to standard therapy.^1, 2^ In order to improve outcome in AML, current efforts in basic and clinical research focus on the genetic characterization of AML in the hopes of furthering our understanding of AML pathogenesis and discovering new targeted therapies that will eventually lead to an increase in the cure rate.^2, 3^ Long non-coding RNAs (lncRNAs), defined as transcripts longer than 200 nucleotides,^4^ affect various cellular functions such as gene regulation, genomic imprinting, RNA maturation and translation.^5, 6^ Moreover, multiple lines of evidence link dysregulation of lncRNAs to diverse human diseases, especially cancer. In the process of carcinogenesis, certain lncRNAs have been attributed to oncogenic and/or tumor suppressor roles.^5^

Maternally expressed gene 3 (*MEG3*) is a myeloid-related lncRNA that has been shown to act as tumor suppressor in solid tumors.^7, 8^ Previous data have demonstrated that *MEG3* is capable of increasing the protein level of the tumor suppressor p53 and enhancing p53 binding to its target promoters.^9^ In consequence, it is very evident that *MEG3* can inhibit tumorigenesis through a p53-dependent manner. However, *TP53* mutations can result in loss of wild-type tumor-suppressing p53 function in diverse types of human cancer, including AML.^10^ *MEG3* has been implicated in the regulation of the RB pathway and thus of cell proliferation,^11, 12^ implying a possible role in a p53-independent pathway. The RB protein can repress gene transcription by directly binding to the transactivation domain of E2F in a complex on the promoters of the E2F target genes including *DNMT3A*.^13^ Accordingly, RB is involved in transcriptional regulation of *DNMT3A* gene in cancer cells,^13^ however, the detailed role of the RB-DNMT3A pathway remains to be fully elucidated.

The Wilms’ tumor 1 (*WT1*) gene, located on chromosome 11p13, encodes a transcriptional regulator that is capable of activating or repressing gene transcription.^14, 15^ The precise role of WT1 in hematopoiesis and its contribution to leukemogenesis are open for speculation. It is reported that *WT1* mutations are associated with an extremely poor outcome,^15^ and they can lead to progression of leukemia by conferring drug resistance.^16, 17^ Dysregulation of the *WT1* gene by epigenetic modifications or mutations might promote leukemic cell proliferation and impair differentiation.^18, 19^ However, the role of WT1 in regulating cancer-related gene expression remains largely unknown, especially whether WT1 controls *MEG3* activity has yet to be explored.

The ten–eleven translocation (TET) family proteins TET1, TET2 and TET3 constitute a novel family of dioxygenases, whose functions are to demethylate DNA sequence by converting 5-methylcytosine to 5-hydroxymethylcytosine.^20^ Pathologically, *TET2* is frequently mutated in hematopoietic malignancies of the myeloid lineage, particularly in AML.^21^ Most recently, WT1 is found to physically interact with TET2 and recruit it to the target genes of WT1,^22^ suggesting that TET2 may be involved in the transcriptional activity of WT1 in AML.

In this study, we demonstrate that inactivation of *MEG3* promotes AML leukemogenesis in a p53-dependent as well as a p53-independent mode. Further analyses show that WT1 specifically binds to the *MEG3* promoter and activates its transcription, and thereby, inducing a reduction of its downstream molecule MDM2. We further investigate the possible association between TET2 and WT1 in AML, and the results suggest that TET2 acts as a cofactor of WT1 to promote *MEG3* transcription. Given the importance of the WT1-*MEG3* axis in suppressing tumor growth, our findings suggest that targeting this axis may represent a novel approach for effective AML treatment.

## Materials and methods

More detailed information on materials and methods can be found in the Supplementary Information.

### Patient and tissue samples

Forty-two AML patient samples were analyzed at the time of diagnosis in this study. AML patients were classified according to French–American–British. All patients gave their written informed consent. The study has been approved by the Ethics Committee of the Institute. Mononuclear cells were isolated by density gradient centrifugation using Lymphoprep, and cryopreserved. In addition, 15 potential donors for allogeneic bone marrow transplantation were used as normal controls. Highly enriched human CD34^+^ cells (>90%) were derived from bone marrow mononuclear cells using MiniMACS (Miltenyi Biotech, Bergisch Gladbach, Germany) following the manufacturer’s instructions. Confirmation of bone marrow-derived CD34^+^ cells phenotype and purity was assessed by immunophenotypic analysis using CD34-FITC (BD Biosciences, San Diego, CA, USA) coupled with flow cytometry. All patients’ samples and controls were provided by the Second Affiliated Hospital of Dalian Medical University.

### Statistical analysis

Student’s *t*-test (two-tailed), *t*-test with Welch's correction, Log-rank (Mantel–Cox) test, *F*-test were performed to analyze the data using GraphPad Prism 6.0 software (GraphPad software Inc., San Diego, CA, USA). *P*-values <0.05 were considered statistically significant. *P<0.05; **P<0.01; ***P<0.001.

## Results

### LncRNA *MEG3* is downregulated in AML

**Figure 1:**
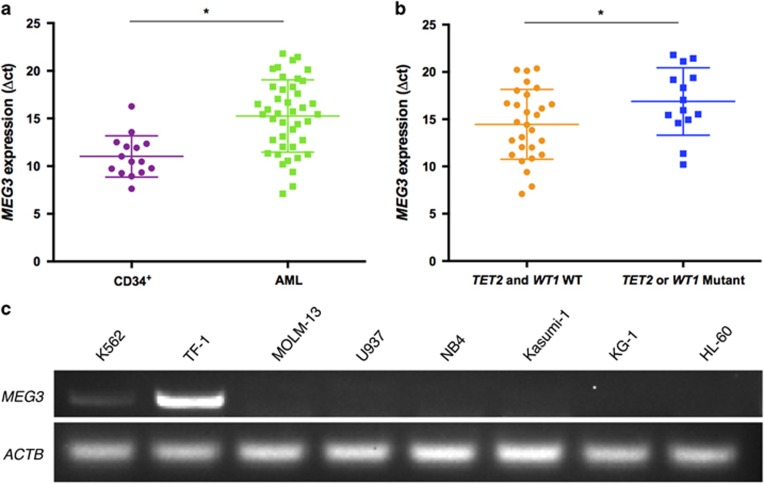
Expression of lncRNA *MEG3* in AML patients and cell lines. (**a**) RT-qPCR analysis of *MEG3* RNA expression in bone marrow of 42 samples from AML patients and CD34^+^ cells derived from 15 potential donors for allogeneic bone marrow transplantation. (**b**) RT-qPCR analysis of *MEG3* RNA expression in bone marrow from AML patients with/without *WT1* or *TET2* mutations. (**c**) PCR analysis of *MEG3* RNA expression in AML cell lines. *P<0.05. RT-qPCR, real-time quantitative PCR.

To gain insights into the patterns of activation of *MEG3* gene in the AML pathogenesis, we examined its expression in normal CD34^+^ bone marrow cells and AML patient samples with different *WT1* or *TET2* mutation status. Given some variations were usually not regarded as true missense mutations (including P29R, I1762V, V218M, L1721W and H1778R) in *TET2*,^23^ only nonsense (4/42, 9.5%) and frameshift mutations (9/42, 21.4%) were used in our analysis. *WT1* mutations (frameshift) were detected in two patients (2/42, 4.8% Supplementary Tables 1 and 2). Real-time quantitative PCR results indicated that *MEG3* was robustly expressed in bone marrow CD34^+^ cells, significantly downregulated in all AML samples, particularly in the *WT1*- or *TET2*-mutant AML subtypes (Figures 1a and b).

For subsequent cell model experiments, we also investigated the genetic backgrounds (Supplementary Tables 1 and 2) and p53 levels (Supplementary Figure 1A) of eight representative cell lines derived from AML (K562, TF-1, MOLM-13, U937, NB4, Kasumi-1, KG-1 and HL-60) and the correlations between *WT1*, *TET2* mutation status/expression levels and *MEG3* inactivation in these cells (Supplementary Figure 1B). As expected, *MEG3* displayed negligible expression in the *WT1*-mutant AML cell lines (U937) relative to *WT1*- and *TET2*-wild-type AML cells (Figure 1c; Supplementary Figure 1B). Taken together, these findings demonstrate that dysregulation of *MEG3* expression definitely correlates with *WT1* or *TET2* mutations, which thus probably plays an important role in AML pathogenesis.

### *MEG3* suppresses tumor growth through a p53-dependent pathway

**Figure 2:**
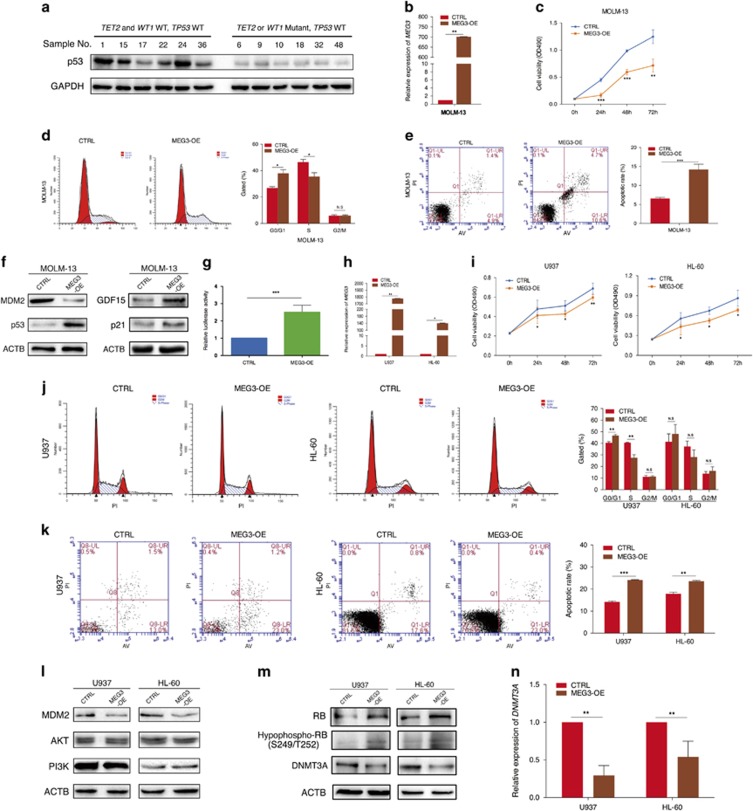
Effect of *MEG3* on cell proliferation and apoptosis *in vitro*. (**a**) Western blotting analysis of p53 protein levels in samples with/without *TET2* or *WT1* mutations. (**b**) RT-qPCR analysis of *MEG3* RNA expression in MOLM-13 cell line. (**c**) MTT assay of the proliferation of MOLM-13 cell line. (**d**) The bar chart represented the percentage of cells in G0/G1, S or G2/M phase, as indicated. (**e**) The apoptotic rates of cells were detected by flow cytometry. (**f**) Western blotting analysis of MDM2, p53, GDF15 and p21 after pCDH-MEG3 and control transfection. (**g**) Induction of *TP53* promoter activity by *MEG3* in MOLM-13 cell line. (**h**) RT-qPCR analysis of *MEG3* RNA expression in U937 and HL-60 cell lines. (**i**) MTT assay of the proliferation of U937 and HL-60 cell lines. (**j**) The bar chart represented the percentage of cells in G0/G1, S or G2/M phase, as indicated. (**k**) The apoptotic rates of cells were detected by flow cytometry. (**l**, **m**) Western blotting analysis of MDM2, AKT, PI3K, RB, hypophospho-RB (S249/T252) and DNMT3A after pCDH-MEG3 and control transfection in U937 and HL-60 cell lines. (**n**) RT-qPCR analysis of *DNMT3A* mRNA expression in U937 and HL-60 cell lines. Results shown were from three independent experiments. ACTB or GAPDH protein was used as an internal control for western blotting analysis. *P<0.05; **P<0.01; ***P<0.001. NS, not significant; RT-qPCR, real-time quantitative PCR.

*MEG3* has been widely demonstrated to exert its biological functions via p53 signaling.^24, 25, 26^ We reasoned that *MEG3* should module AML pathogenesis to some extent in a similar way. On the basis of the data of genetic status of AML samples (Supplementary Tables 1 and 2), we discovered that p53 protein levels were dramatically reduced in samples with *TET2* or *WT1* mutations (Figure 2a). Then we examined the regulatory roles of *MEG3* in cellular phenotypes and key signaling molecules in *TP53wt* AML cell line. Overexpression of *MEG3* in MOLM-13 (*WT1wt, TET2wt*and*TP53wt*) cell line was found to significantly suppress cell proliferation and induce G0/G1 cell cycle arrest and apoptosis when compared with controls (Figures 2b–e). In addition, *MEG3* was found to positively regulate the expression of p53 and p53 target genes (including *p21* and *GDF15*, but has no effect on *BAX*, *NOXA* and *PUMA*), and negatively regulate MDM2 expression (Figure 2f; Supplementary Figure 2A). Furthermore, luciferase reporter assays confirmed the p53 transcriptional activity in the MOLM-13 cells (Figure 2g). Intriguingly, forced expression of *MEG3* had no obvious effect on PI3K, AKT, RB and hypophosphorylated RB (S249/T252) expression levels (Supplementary Figure 2B).

### *MEG3* suppresses tumor growth through a p53-independent pathway

Given that *TP53* mutations or loss are frequently detected in patients with therapy-related AML or AML with complex karyotype,^27, 28^ we therefore next explored whether *MEG3* plays a role as tumor suppressor in AML cell lines absent of *TP53*. As in the *TP53wt* AML cell line, stable overexpression of *MEG3* in the two *TP53mut* AML cell lines U937 (*WT1mut, TET2wt*) and HL-60 (*WT1wt, TET2wt*) significantly suppressed cell proliferation, induced G0/G1 cell cycle arrest and apoptosis when compared to controls (Figures 2h–k). In contrast, knockdown of *MEG3* in K562 (*WT1wt, TET2wt*) and TF-1 (*WT1wt, TET2wt*) cell lines resulted in a significant increase in cell proliferation, a failure to restrict cell cycle progression and induce apoptosis (Supplementary Figures 2C–F). These results suggest that *MEG3* also suppresses tumor growth through a p53-independent pathway.

Subsequently, we interrogated the molecular mechanism by which *MEG3* negatively regulates cell proliferation in a p53-independent manner by examining the expression of MDM2, PI3K, AKT and RB. Gain- or loss-of-function experiments in the above selected AML cell lines revealed that *MEG3* negatively regulates MDM2 at the protein expression level (Figure 2l; Supplementary Figure 2G). Although the changes in AKT and PI3K expression were not obvious, RB and hypophosphorylated RB (S249/T252) protein levels correlated positively with *MEG3* abundances (Figures 2l and m; Supplementary Figures 2G and H).

Extensive studies have suggested that RB functions as a classic tumor suppressor and inhibits cell proliferation mainly by negatively regulating the transcription of certain genes that are required for cell cycle progression. Depletion of MDM2 increases the total RB and the active form of hypophosphorylated RB at S249/T252.^13^ RB lacks a DNA-binding domain and is tethered to promoters through its interaction with other sequence-specific transcription factors such as members of the E2F family of proteins. RB binds to the transcription activation domain of E2F and blocks its activity. More importantly, the RB–E2F complex also actively represses transcription on promoters that contain E2F sites.^29^ Indeed, *DNMT3A* is one of genes whose promoters contain E2F sites. To examine whether *MEG3* affects the expression of *DNMT3A* by promoting hypophosphorylation of RB at S249/T252, we determined the expression levels of DNMT3A in AML cells after silencing or overexpressing *MEG3*. The results showed that both mRNA and protein expression levels of DNMT3A negatively correlated with *MEG3* abundances (Figures 2m and n; Supplementary Figures 2H and I). Taken together, these data demonstrate that *DNMT3A* is a *MEG3* downstream gene and that *MEG3* probably plays crucial roles in inhibiting tumor growth by downregulating *DNMT3A* via the MDM2/RB signaling pathway in AML cells in the absence of p53.

### *MEG3* inhibits AML leukemogenesis *in vivo*

**Figure 3:**
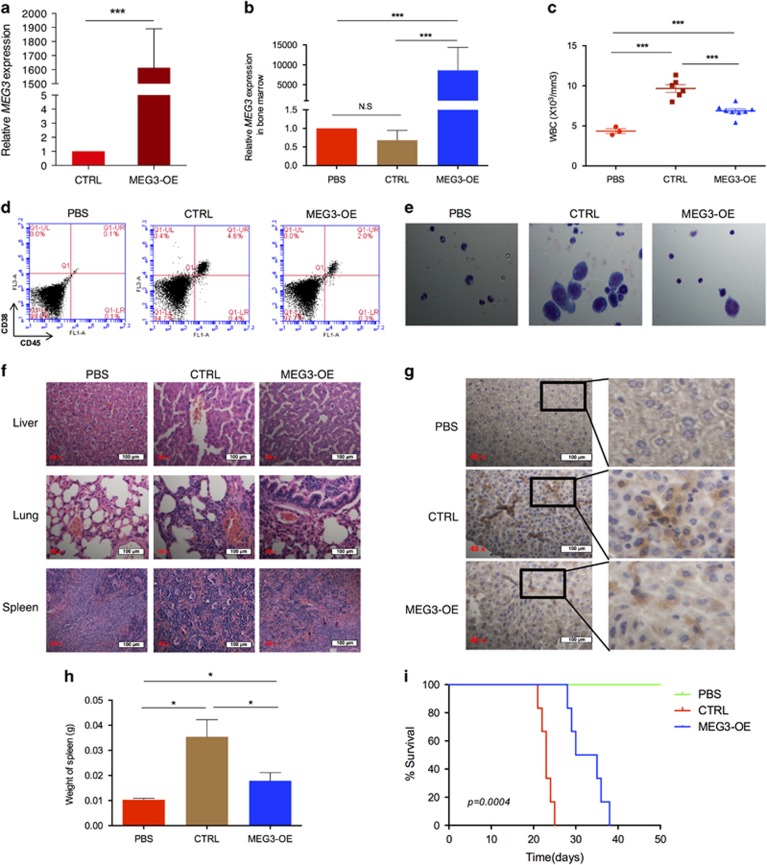
*MEG3* inhibits AML leukemogenesis *in vivo*. (**a**) Relative *MEG3* RNA expression in U937 cells stably transfected with CTRL and MEG3-OE. (**b**) Relative *MEG3* RNA expression in bone marrow of PBS (*n*=3), U937-CTRL (*n*=6) and U937-MEG3-OE (*n*=8) at 4 weeks. (**c**) Peripheral blood WBC count at 4 weeks. (**d**) CD45^+^ and CD38^+^ immunophenotype of PBS, U937-CTRL and U937-MEG3-OE treated for 4 weeks. (**e**) Bone marrow morphology in PBS, U937-CTRL and U937-MEG3-OE. Scale bar, 10 μm. (**f**) H&E of liver, lung and spleen of PBS, U937-CTRL and U937-MEG3-OE at 4 weeks. Scale bars represented 100 μm. (**g**) IHC of liver with CD45 antibody of PBS, U937-CTRL and U937-MEG3-OE at 4 weeks. (**h**) The weight of spleen of mice treated with PBS, U937-CTRL and U937-MEG3-OE. (**i**) Kaplan–Meier survival curve of PBS, U937-CTRL and U937-MEG3-OE. *P<0.05; ***P<0.001. H&E, hematoxylin and eosin; IHC, immunohistochemistry; NS, not significant; PBS, phosphate-buffered saline; WBC, white blood cells.

To investigate whether *MEG3* affects AML leukemogenesis *in vivo*, two types of mouse models were established. We first examined the effects of *MEG3* on the robust engraftment of AML in NOD-SCID mice. U937 cells transfected with pCDH-MEG3 (MEG3-OE) or control vector (CTRL; Figure 3a) were injected into 6–8-week-old mice. Four weeks after tail injection, the mice transplanted with U937-CTRL showed higher frequencies of serious paralysis and accumulation of urine in bladders than those with U937-MEG3-OE (Supplementary Figure 3A). After U937-MEG3-OE injection, *MEG3* was highly expressed in the peripheral blood and bone marrow, and the number of white blood cells decreased dramatically (Figures 3b and c; Supplementary Figure 3B). Bone marrow assay showed that a significant decrease in CD45^+^/CD38^+^ cells in mice injected with U937-MEG3-OE, indicating that *MEG3* inhibits the percentage of engraftment (Figure 3d). A large amount of monocelluar cells with megakaryocytes was observed in AML mice (Figure 3e). Leukemic infiltration was also observed in multiple organs after robust NOD-SCID engraftment of numerous AML cells (Figures 3f and g; Supplementary Figure 3C). We also detected the more serious splenomegaly in mice injected with U937-CTRL compared with U937-MEG3-OE (Figure 3h; Supplementary Figure 3D). Furthermore, our data reveal that *MEG3* expression prolongs the survival of AML mice (Figure 3i).

We next injected U937 cells transfected with pCDH-MEG3 or control into female athymic nude (nu/nu) mice. Twenty-one days after the injection, we observed that the tumors formed in the U937-MEG3-OE group were substantially smaller compared to those from the U937-CTRL group (Supplementary Figures 3E and F). The average tumor weights were markedly lower in the U937-MEG3-OE group than the control group at the end of the experiment (Supplementary Figure 3G). As predicted, the expression levels of *MEG3* in tumor tissues of the U937-MEG3-OE group were significantly higher than those of the control group (Supplementary Figure 3H). Moreover, hematoxylin and eosin and immunohistochemistry showed that proliferating cell nuclear antigen levels in tumor tissues of the U937-MEG3-OE group exhibited decreased positivity for proliferating cell nuclear antigen than in those of the U937-CTRL group (Supplementary Figures 3I and J). Taken together, these results suggest that *MEG3* limits the proliferation of AML cells *in vivo*.

### *MEG3* is transcriptionally regulated by WT1 in AML

**Figure 4:**
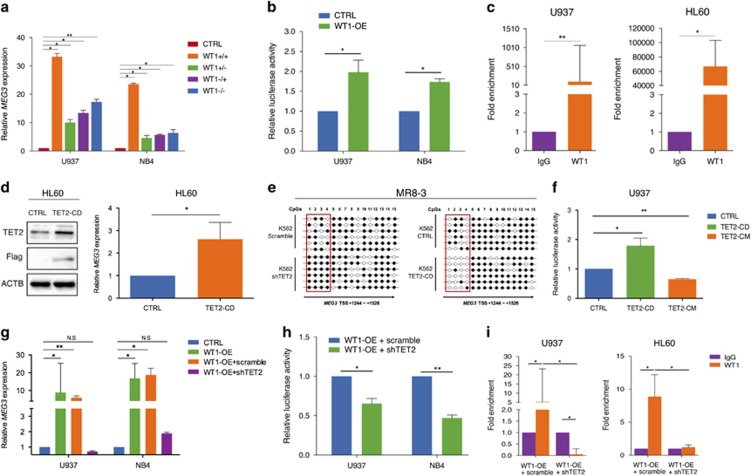
WT1 interacts with TET2 to co-regulate *MEG3* expression in AML cells. (**a**) RT-qPCR analysis of *MEG3* RNA expression after transfected with four major *WT1* splicing variants (+5/+KTS, +5/−KTS, −5/+KTS, −5/−KTS) in U937 and NB4 cell lines. (**b**) Induction of *MEG3* promoter activity by WT1 in U937 and NB4 cell lines. (**c**) The WT1 binding at the promoter regions of *MEG3* was assessed by ChIP analysis. (**d**) HL-60 cell line was transfected with a Flag-TET2^CD^ construct, western blotting analysis of TET2 and Flag, RT-qPCR analysis of *MEG3* RNA expression. (**e**) A representative methylation pattern of the CpGs in K562 cells after bisulfite treatment. Each line represented one PCR product, and six PCR products were shown for each sample. (**f**) Induction of *MEG3* promoter activity by *TET2*^CD^ or *TET2*^CM^ in U937 cells. (**g**) WT1 was transiently overexpressed either singly or with shRNA against *TET2* in U937 and NB4 cells, RT-qPCR was examined for the RNA expression of *MEG3*. (**h**) Induction of *MEG3* promoter activity by WT1 either singly or with shRNA against *TET2* in U937 and NB4 cell lines. (**i**) WT1 was transiently overexpressed either singly or shRNA against *TET2* in U937 and HL-60 cells. The WT1 binding at the promoter regions of *MEG3* was assessed by ChIP analysis. Representative images of three independent experiments were shown. ACTB protein was used as an internal control for western blotting analysis.*P<0.05; **P<0.01. •, methylated CpG; ○, unmethylated CpG; ChIP, chromatin immunoprecipitation; NS, not significant; RT-qPCR, real-time quantitative PCR; shRNA, short hairpin RNA.

*WT1* mutations or inactivation have been implicated in the leukemogenesis of AML.^30^ The correlation between WT1 and *MEG3* expression in clinical samples and AML cell lines therefore led us to hypothesize that the WT1 transcription factor regulates *MEG3* expression. To test this conjecture, we performed *in silico* analysis^31^ on the *MEG3* promoter sequence and located a binding site of WT1 in this region (Supplementary Figure 4A). Relatively low-expression levels of WT1 were detected in U937 and NB4 cells (Supplementary Figures 4B and C), but a significant increase in *MEG3* expression levels was observed following the transfection of wt *WT1* into the two cell lines (Figure 4a). In contrast, *WT1* depletion dramatically decreased *MEG3* expression in KG-1 cells (*WT1wt*, *TET2wt*) (Supplementary Figures 4D and E). To examine whether WT1 can activate the transcription of *MEG3*, we constructed a *WT1* luciferase reporter by inserting the sequence of *MEG3* promoter region into the pGL4 vector. As shown in Figure 4b, compared with controls, the luciferase reporter assays revealed that overexpression of *WT1* induced the transcriptional activation of *MEG3*, whereas knockdown of *WT1* significantly reduced its transcription activity (Supplementary Figures 4F). To verify the prediction for the WT1 binding site, we performed chromatin immunoprecipitation assay in U937 and HL-60 cell lines using WT1-specific antibody after transfection of wt *WT1* into the two cell lines. The chromatin immunoprecipitation results confirmed the direct binding of WT1 on the endogenous *MEG3* promoter (Figure 4c). These findings provide compelling evidence that WT1 binds to *MEG3* promoter and thus activates *MEG3* transcription.

### *MEG3* expression is associated with its first intron methylation mediated by TET2

Previous study has demonstrated that *MEG3* gene expression is under epigenetic control, and aberrant CpG methylation occurs in AML patients.^32^ Downregulation of *MEG3* in our *TET2*-mutant AML subtype samples implies that *TET2* is likely to modulate *MEG3* expression. We found that overexpression of *TET2* CD domain (*TET2*^*CD*^) that contains both the Cys-rich and the DSBH domains could lead to the upregulation of *MEG3* expression (Figure 4d), whereas depletion of *TET2* resulted in the downregulation of *MEG3* (Supplementary Figures 5A and B). Furthermore, we confirmed that the catalytic domain was required for the effect of TET2 on activating *MEG3* expression, as the TET2 catalytic inactive mutant (*TET2*^*CM*^) could not upregulate *MEG3* expression (Supplementary Figures 5C and D). DNA methylation is a common mechanism for promoter repression, and it also contributes to inactivation of enhancer elements usually existing in regions outside of promoters, particularly in introns. It is also noteworthy that expression of the *MEG3*-DLK1 locus may be regulated by differentially methylated regions (DMRs). Therefore, the methylation status of multiple *cis* elements (including promoter, enhancer, DMR and the first intron) of *MEG3* gene was assayed to determine whether *TET2* affects the expression of *MEG3* through the epigenetic mechanism. Interestingly, after knockdown or overexpression of *TET2* in K562 cells, no apparent changes in the DNA methylation levels were detected in the promoter, enhancer and DMR regions of *MEG3*, but the significant changes were observed in the first intron (Figure 4e; Supplementary Figures 6A and B). The same results were observed in HL-60 cell line (Supplementary Figure 6C). In agreement, the luciferase reporter assays showed that overexpression of *TET2*^*CD*^, but not the *TET2*^*CM*^, induced the transcriptional activity of the *MEG3* promoter in U937 cells (Figure 4f). Collectively, these results suggest that TET2 can activate *MEG3* transcription, which is associated with the ability of TET2 to mediate the *MEG3* first intron DNA methylation.

### The transactivation activity of WT1 on *MEG3* is modulated by TET2 in AML

As described above, both WT1 and TET2 affect the transcriptional activity of *MEG3*. Both of them are found to be mutated, which are believed to play key roles in AML leukemogenesis. However, their mutations are mutually exclusive, indicating they may function in the same pathway to suppress AML. Moreover, WT1 has recently been demonstrated to physically bind and recruit TET2 to its target genes.^22^ This led us to propose that TET2 may serve as a cofactor of WT1 in its regulation of *MEG3* transcription, as TET2 lacks a DNA-binding domain. To validate this idea, we first determined the WT1–TET2 association by coupled immunoprecipitation and western blotting (IP-western) in NB4 and 293 T-cell lines. As shown in Supplementary Figure 7A, we demonstrated that the WT1–TET2 interaction could readily be detected in the two cell lines. In addition, *WT1* was transiently expressed either alone or with shTET2 in U937 and NB4 cell lines. The cells only transfected with the *WT1* vector alone expressed higher *MEG3* levels than those transfected with both the *WT1* and shTET2 vectors (Figure 4g; Supplementary Figures 7B and C). To elucidate the influence of the WT1 and TET2 interaction on the transcriptional activity of *MEG3*, the luciferase assay was carried out to test the activity of *MEG3* promoter. Figure 4h showed that cells transfected only with the *WT1* vector exhibited higher luciferase activity than those transfected simultaneously with the *WT1* and shTET2 vectors. Moreover, silencing of *WT1* expression with short hairpin RNA together with transfection with *TET2*^*CD*^ vector in KG-1 cells resulted in higher luciferase activities compared with *WT1* short hairpin RNA vector only (Supplementary Figure 7D). Subsequently, chromatin immunoprecipitation and real-time quantitative PCR assays were used to further explore whether regulation of *MEG3* promoter by WT1 requires TET2. Consistently, WT1 was found to be less enriched on the *MEG3* promoter in cells simultaneously transfected with *WT1* and shTET2 vectors than those transfected only with *WT1* vector (Figure 4i). Taken together, these results suggest that TET2 serves as a cofactor for WT1 in the regulation of *MEG3* transcriptional activation in AML cells.

## Discussion

Mounting evidence has supported the notion that lncRNAs are involved in tumorigenesis.^7^ LncRNA *MEG3*, silenced in various types of cancer, is commonly regarded as a tumor suppressor. Although *MEG3* low expression and promoter hypermethylation have been found to be markers of poor prognosis in AML patients,^33^ the functional consequence of *MEG3* downregulation and underlying mechanisms involved have remained elusive. In this study, we show that *MEG3* suppresses AML leukemogenesis through both p53-dependent and p53-independent pathways. More importantly, our work reveals *MEG3* as a novel target of WT1 and that TET2 interacts with WT1 to co-regulate *MEG3* transcriptional activation.

Our works show that ectopic expression of *MEG3* suppresses cell proliferation and induces G0/G1 cell cycle arrest in AML, consistent with previous observations in other solid tumors.^24, 25, 26, 34^ The inhibitory roles of *MEG3* in AML leukemogenesis are further verified in two independent *in vivo* mouse models. It is well-known that *MEG3* protects the p53 from degradation, and thus inhibits tumor growth in a p53-dependent fashion.^35^ Our experiments extend our understanding of the molecular events leading to AML leukemogenesis, where decreased expression of *MEG3* significantly restricts the increase of the tumor suppressor p53 protein.^34, 35, 36^ These observations often further support for a common tumor suppressive mechanism, whereby *MEG3* functions as a tumor suppressor, at least partly via the activation of p53 in multiple cancers.

*MEG3* is capable of inducing MDM2 degradation, thereby stabilizing the p53 protein. However, p53 is often lost or mutated in patients including therapy-related AML or AML with complex karyotype.^27, 28^ It has been demonstrated that MDM2 contributes to tumor initiation and progression even when p53 is no longer active.^37^ Our data confirm that *MEG3* inhibits AML cell growth under genetic backgrounds of *TP53* mutation, deletion or depletion, by decreasing MDM2 protein level. We discover that MDM2 functionally interacts with its target RB in AML. *MEG3* promotes MDM2 degradation, and eventually increases the total RB level and the active form of hypophosphorylated RB.^38, 39^ After activation, RB interacts with transcription factor E2F to negatively regulate expression of their target genes. We uncover that *DNMT3A*, a very important target of E2F complex, is markedly downregulated by *MEG3*. Given the crucial role of DNMT3A in AML, it is most probably that *MEG3* inhibits tumor growth through RB-DNMT3A pathway. Apart from the p53-dependent pathway, our results unveil a novel p53-independent mechanism by which *MEG3* exerts its tumor-suppressive function in AML.

Currently, the observations that WT1 is expressed in the majority of AML cases, and that it is mutated in a proportion of AMLs have led to a number of studies to decipher the mechanistic role of WT1.^30^ The *WT1* mutations clustering in exons 7 and 9 are correlated with lower complete remission rates, higher relapse rates, shorter disease-free survival and overall survival.^15, 19^ The specific genes that are regulated by WT1 and become deregulated by *WT1* mutation remain an area of intense investigation.^30^ Our findings that WT1 positively modulates the transcription of *MEG3* provide an explanation for the mechanism of leukemogenesis in WT1-mutant AML.

It is long known that promoter hypermethylation is a common mechanism contributing to transcriptional repression.^40^ Moreover, DNA methylation has been found to correlate negatively with enhancer activity and gene expression, possibly by interfering with transcription factor binding.^41^ These functionally relevant binding sites for transcription factors, however, usually exist in regions outside of promoters, particularly in introns.^42^ Gene expression is usually regulated by the methylation status of DNA *cis* elements such as promoters, enhancers and introns.^43^ It is also noteworthy that expression of the *MEG3*-DLK1 locus may be regulated by DMRs.^44, 45^ Our results show that the promoter, enhancer, DMR and the first intron of *MEG3* display aberrant methylation, consistent with others findings.^32, 46^ However, only the first intron displays significant change in methylation levels upon TET2 expression, meaning that the methylation of this region might be in a dynamic state. Differential methylation at intronic enhancers has been previously reported to affect gene expression.^47, 48, 49^ Our results show that the induction of *MEG3* expression by TET2 is associated with methylation of its first intron. Collectively, these results suggest the presence of putative regulatory elements in the first intron of *MEG3* and *MEG3* expression may thus be repressed by hypermethylation as a result of lack of functional TET2, contributing to *MEG3* epigenetic silencing occurred specifically in the *TET2*-mutant AML subtype.

*TET2* is able to inhibit leukemogenesis, but it is frequently mutated in AML. *TET2* is found to be mutated in a mutually exclusive manner with *WT1*, indicating that they both may suppress AML through the same pathway. Most recently, WT1 has been demonstrated to physically interact with TET2 and recruit it to the target genes of WT1,^22^ implying that TET2 may be involved in modulating the transcriptional activity of *WT1*. As expected, our data confirm that WT1 interacts directly with TET2, and we further demonstrate that TET2 serves as a cofactor of WT1 to activate *MEG3* expression. Not surprisingly, the mutation of TET2 substantially diminishes the transaction activity of WT1 on *MEG3*. Taken together, these results suggest there exists a linear TET2-WT1-*MEG3* axis in the suppression of AML leukemogenesis, and therefore dysfunction of this axis; such mutations to either *TET2* or *WT1* may be a key contributor to AML. Evidently, our study uncovers a novel mechanism by which WT1–TET2 plays a critical role through the regulation of *MEG3* gene expression.

In summary, our work demonstrates that the loss of lncRNA *MEG3* leads to AML leukemogenesis, indicating that its overexpression can suppress AML development via both p53-dependent and p53-independent pathways. Furthermore, the discovery that WT1 cooperates with TET2 to upregulate *MEG3* expression places TET2-WT1-*MEG3* signaling axis as a central tumor suppressive pathway in AML, therefore emphasizing the potentials of this axis in AML diagnosis and therapy.
