# Supplementary material for: Dysfunction of the WT1-MEG3 signaling promotes AML leukemogenesis via p53-dependent and -independent pathways
Source: Leukemia. 2017 May 2;31(12):2543–51. doi: 10.1038/leu.2017.116 (PMC5729340; doi:10.1038/leu.2017.116)
Supplement: Supplementary Information [file leu2017116x1.docx]

**Supplementary Data**

**Dysfunction of the WT1-*MEG3* Signaling Promotes AML Leukemogenesis via p53 Dependent and Independent Pathways**

Yizhu Lyu^1, 2#^, Jiacheng Lou,^1, 3#^, Yan Yang^1, 2#^, Jiuxing Feng^1#^, Yuchao Hao^1^, Shuyu Huang^1^, Linlin Yin^1, 2^, Jiangbing Xu^1^, Dan Huang^1, 2^, Binbin Ma^1, 3^, Deyu Zou^1^, Yue Wang^1, 2^, Yue Zhang^1^, Bo Zhang^3^, Puxiang Chen^4^, Kanglun Yu^5^, Eric W.-F. Lam^6^, Xiang Wang^1*^, Quentin Liu^1*^, Jinsong Yan^1, 2*^, Bilian Jin^1*^

^1^Department of Hematology, the Second Affiliated Hospital; Institute of Cancer Stem Cell, Cancer Center, Dalian Medical University, Dalian 116001, Liaoning, P.R. China.

^2^ Institute of Hematopoeitic Stem Cell Transplantation of Dalian Medical University;

Liaoning Hematopoeitic Stem Cell Transplantation Medical Center, Dalian Key Laboratory of Hematology, Department of Hematology of the Second Affiliated Hospital of Dalian Medical University, Dalian 116001, Liaoning, P.R. China.

^3^Department of Neurosurgery, the Second Affiliated Hospital of Dalian Medical University, Dalian 116001, Liaoning, P.R. China.

^4^Department of Obstetrics and Gynecology, the Second Xiangya Hospital, Central South University, Changsha 410011, Hunan, P.R. China.

^5^Department of Cellular Biology and Anatomy, Augusta University, Augusta, GA, USA.

^6^Department of Surgery and Cancer, Imperial College London, London W12 0NN, UK.

^#^ These authors contributed equally to this work

**Running Title:** WT1-TET2 Complex Regulates LncRNA *MEG3* in AML

***Corresponding author:** Prof. Bilian Jin, Tel and Fax: +86 (411) 86110530, e-mail: jinbilian@dmu.edu.cn; Prof. Jinsong Yan, e-mail:  [yanjsdmu@126.com](mailto:yanjsdmu@126.com); Prof. Quentin Liu, e-mail: [liuq9@mail.sysu.edu.cn](mailto:liuq9@mail.sysu.edu.cn); Dr. Xiang Wang, e-mail: [wangx6281@dmu.edu.cn](mailto:wangx6281@dmu.edu.cn).

**Supplementary Figures**

**Supplementary Figure 1.** (A)Western blotting analysis of p53 protein levels in AML cell lines. (B) RT-qPCR analysis of *MEG3* RNA expression in *WT1*- and *TET2*-wild type AML cell lines (K562, TF-1, MOLM-13, NB4, Kasumi-1, KG-1 and HL-60) and *WT1*-mutant (U937) cell line. GAPDH protein was used as an internal control for Western blotting analysis.

**Supplementary Figure 1**

**
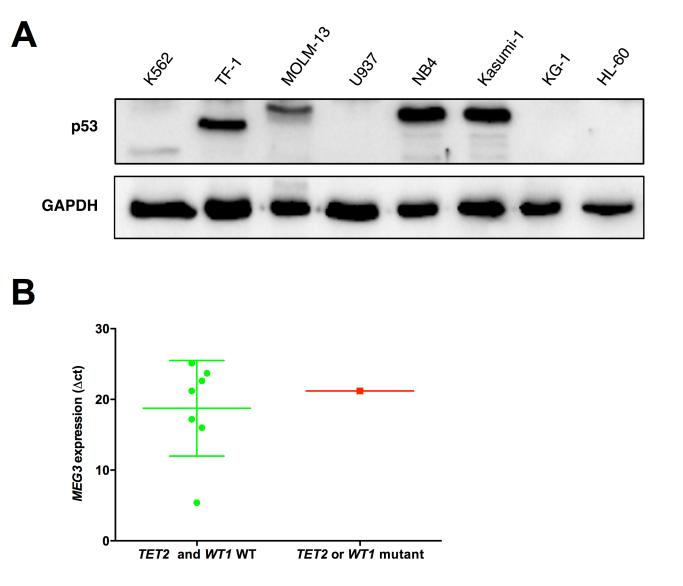
**

**Supplementary Figure 2.** (A，B) Western blotting analysis of p53 targets (including BAX, NOXA, and PUMA), PI3K, AKT, RB and hypophospho-RB (S249/T252) in MOLM-13 cell line. (C) RT-qPCR analysis of *MEG3* RNA expression in K562 and TF-1 cell lines after transfection of MEG3 siRNAs. (D) MTT assay of the proliferation of K562 and TF-1 cell lines after transfection of MEG3 siRNA-1. (E) The bar chart represented the percentage of cells in G0/G1, S, or G2/M phase, as indicated. (F) The apoptotic rates of cells were detected by flow cytometry. (G，H) Western blotting analysis of MDM2, AKT, PI3K, RB, hypophospho-RB (S249/TS252) and DNMT3A after transfection of MEG3 siRNA-1 in K562 and TF-1 cell lines. (I) RT-qPCR analysis of *DNMT3A* mRNA expression in K562 and TF-1 cell lines. Results shown were from 3 independent experiments. ACTB protein was used as an internal control for Western blotting analysis. **p* < 0.05; ***p* < 0.01; ****p* < 0.001.

**Supplementary Figure 2**

**
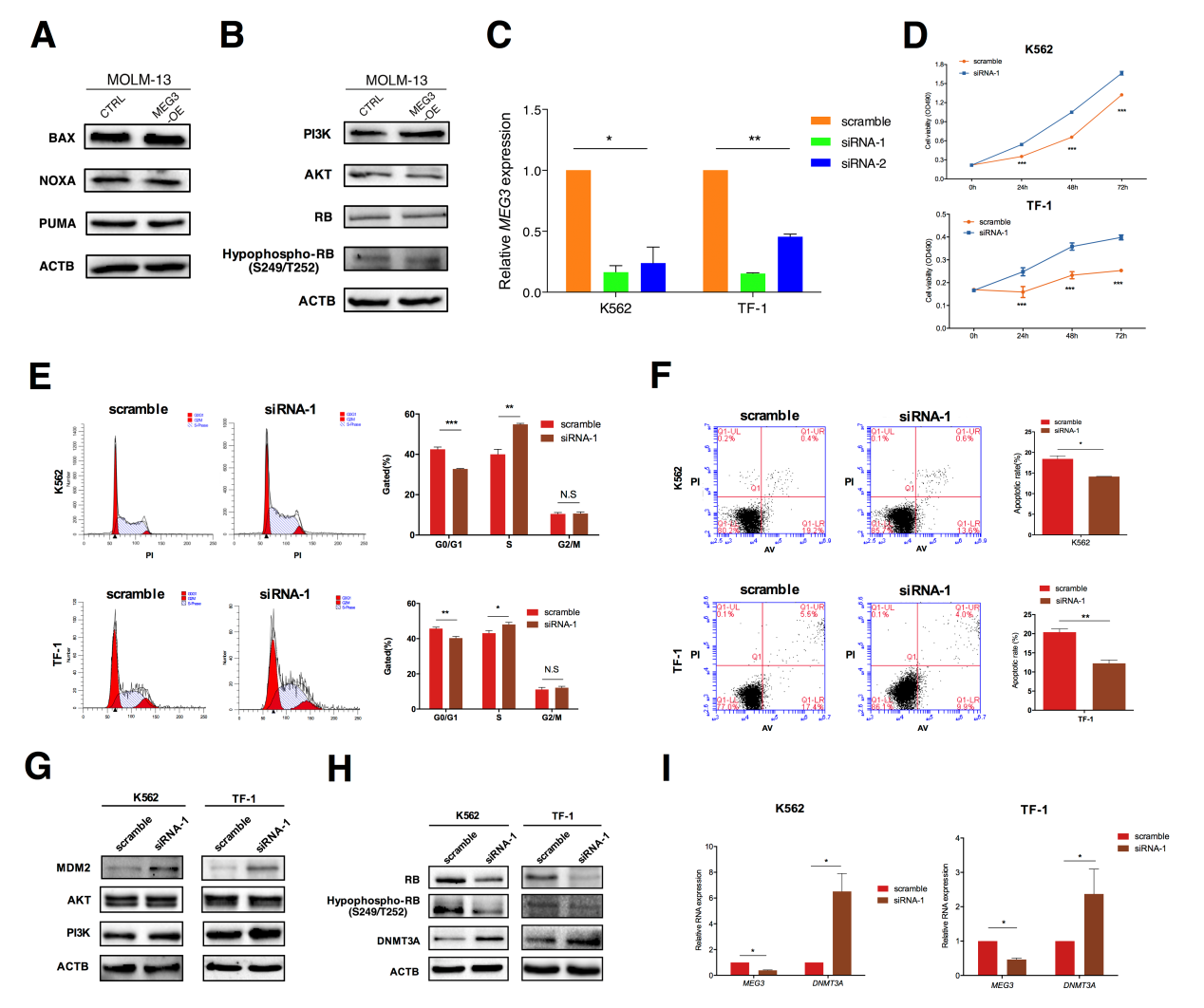
**

**Supplementary Figure 3.** (A) Accumulation of urine in bladder of AML mice at 4 weeks after injected tumor cells. (B) Relative *MEG3* RNA expression in peripheral blood of mice. (C) IHC of lung and spleen with CD45 antibody of mice treated with PBS, U937-CTRL or U937-MEG3 cell line for 4 weeks. (D) The photograph of spleen of AML mice. (E，F) The tumor volume was calculated once every three days after injection of U937 cells stably transfected with pCDH-MEG3 or control. Points, mean (n = 5); bars indicated S.D. (G) Tumor weights were represented as means of tumor weights±S.D. (H) RT-qPCR analysis of *MEG3* expression in tumor tissues formed from U937-CTRL or U937-MEG3. (I，J) H&E and IHC of tumors developed from pCDH-MEG3 transfected U937 cells showed lower PCNA protein levels than tumors developed by control cells. **p* < 0.05; ****p* < 0.001; N.S, not significant.

**Supplementary Figure 3**

**
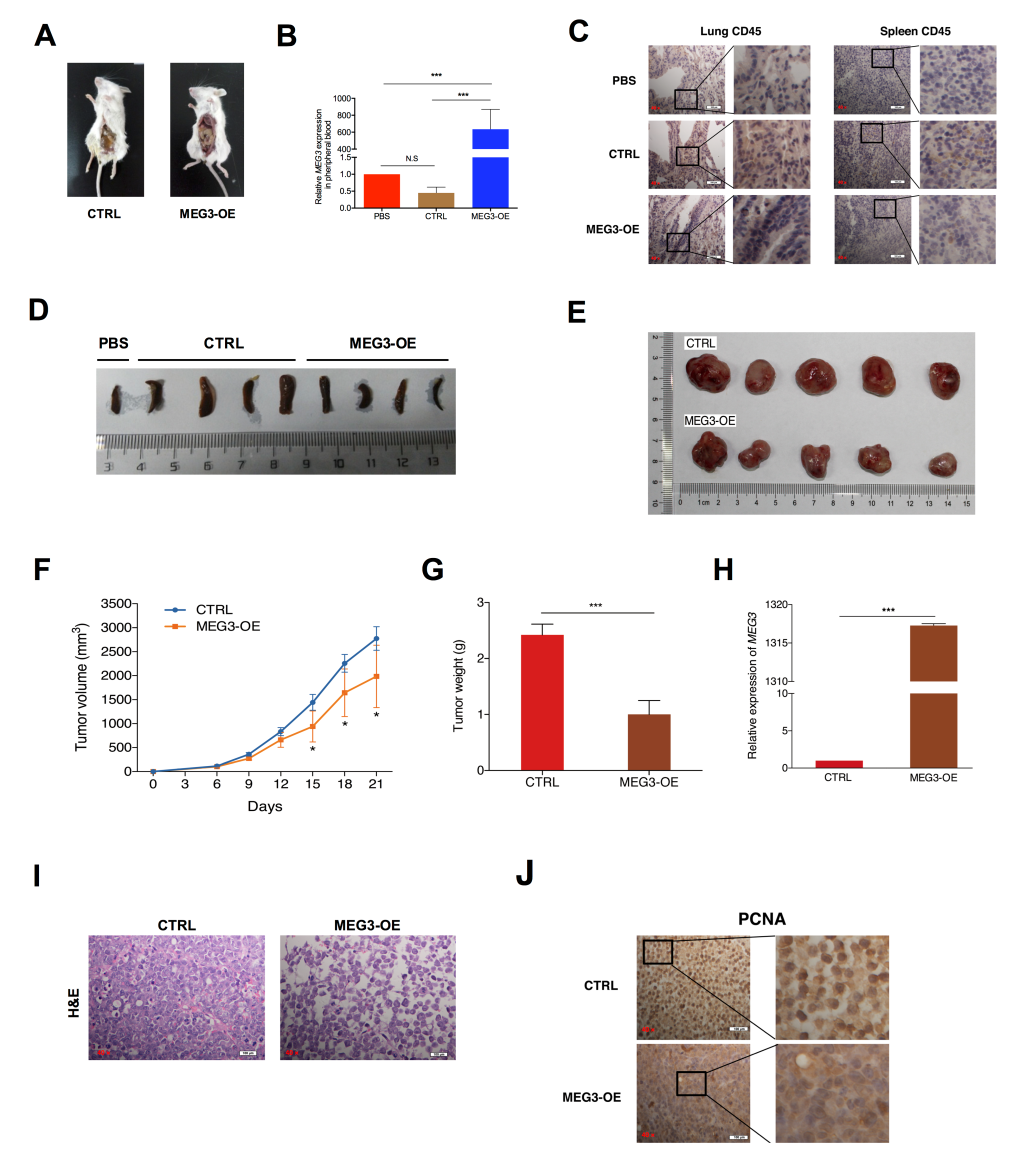
**

**Supplementary Figure 4.** (A) Description of WT1-binding sites in promoter region of *MEG3*. (B，C) Western blotting and RT-qPCR analysis of WT1 expression after transfected with four major *WT1* splicing variants (+5/+KTS, +5/−KTS, −5/+KTS, −5/−KTS) in U937 and NB4 cell lines. (D) Western blotting analysis of WT1 expression after transfected with shRNAs against WT1 in KG-1 cells. (E) RT-qPCR analysis of *WT1* and *MEG3* RNA expression in KG-1 cells after transfected with shRNAs against *WT1*. (F) Reduction of *MEG3* promoter activity by shWT1-1 in KG-1 cells. Shown were representative images of three independent experiments. ACTB protein was used as an internal control for Western blotting analysis. **p* <0.05; ***p* < 0.01; ****p* < 0.001.

**Supplementary Figure 4**

**
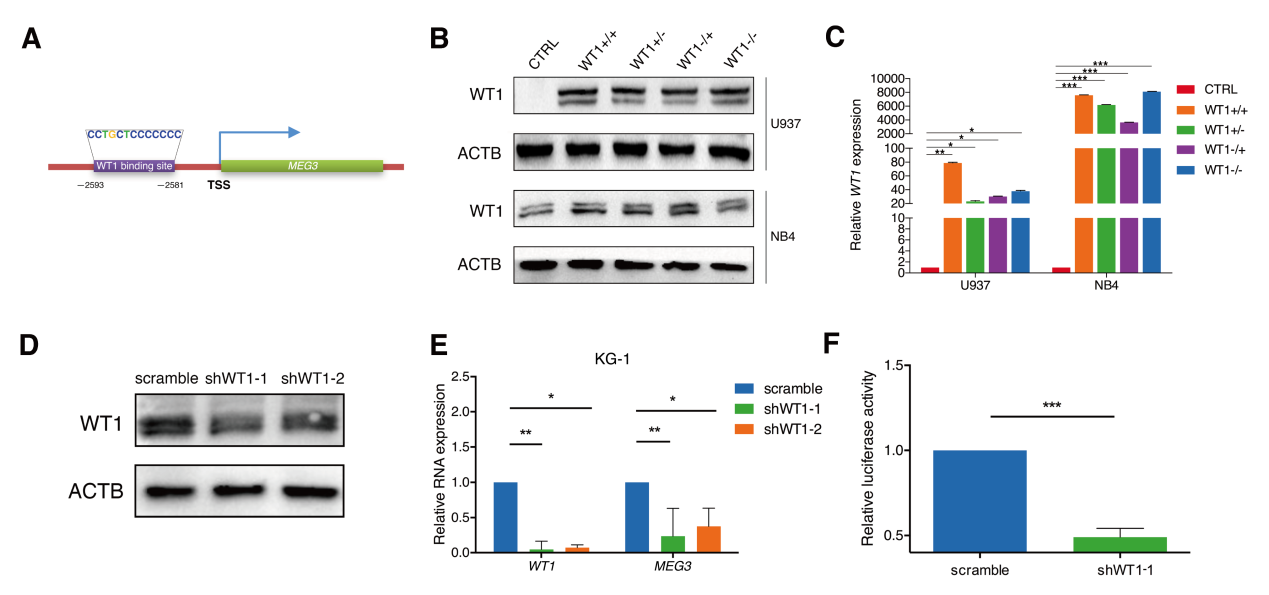
**

**Supplementary Figure 5.** (A, B) K562 cells were transfected with a shRNA against *TET2*, Western blotting analysis of TET2 and ACTB, RT-qPCR analysis of *MEG3* RNA expression. (C, D) K562 cells were transfected with a Flag-TET2^CM^ construct, Western blotting analysis of TET2, Flag and ACTB, RT-qPCR analysis of *MEG3* RNA expression. Shown were representative images of three independent experiments. ACTB protein was used as an internal control for Western blotting analysis. **p* < 0.05; ****p* < 0.001; N.S, not significant.

**Supplementary Figure 5**


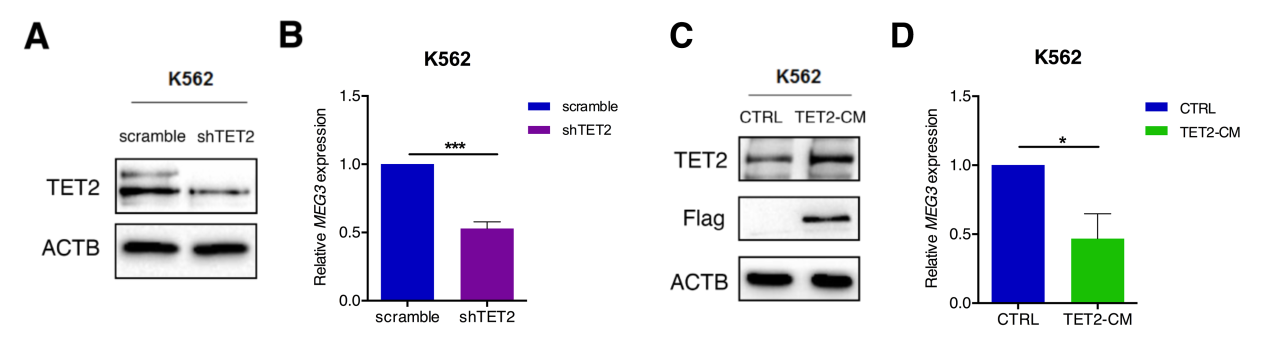


**Supplementary Figure 6.** (A) Description of MEG3 methylation region. (B，C) A representative methylation pattern of the CpGs in K562 and HL-60 cell lines after bisulfite treatment. Each line represented one PCR product, and four PCR products were shown for each sample. ●, Methylated CpG; ○, unmethylated CpG.

**Supplementary Figure 6**


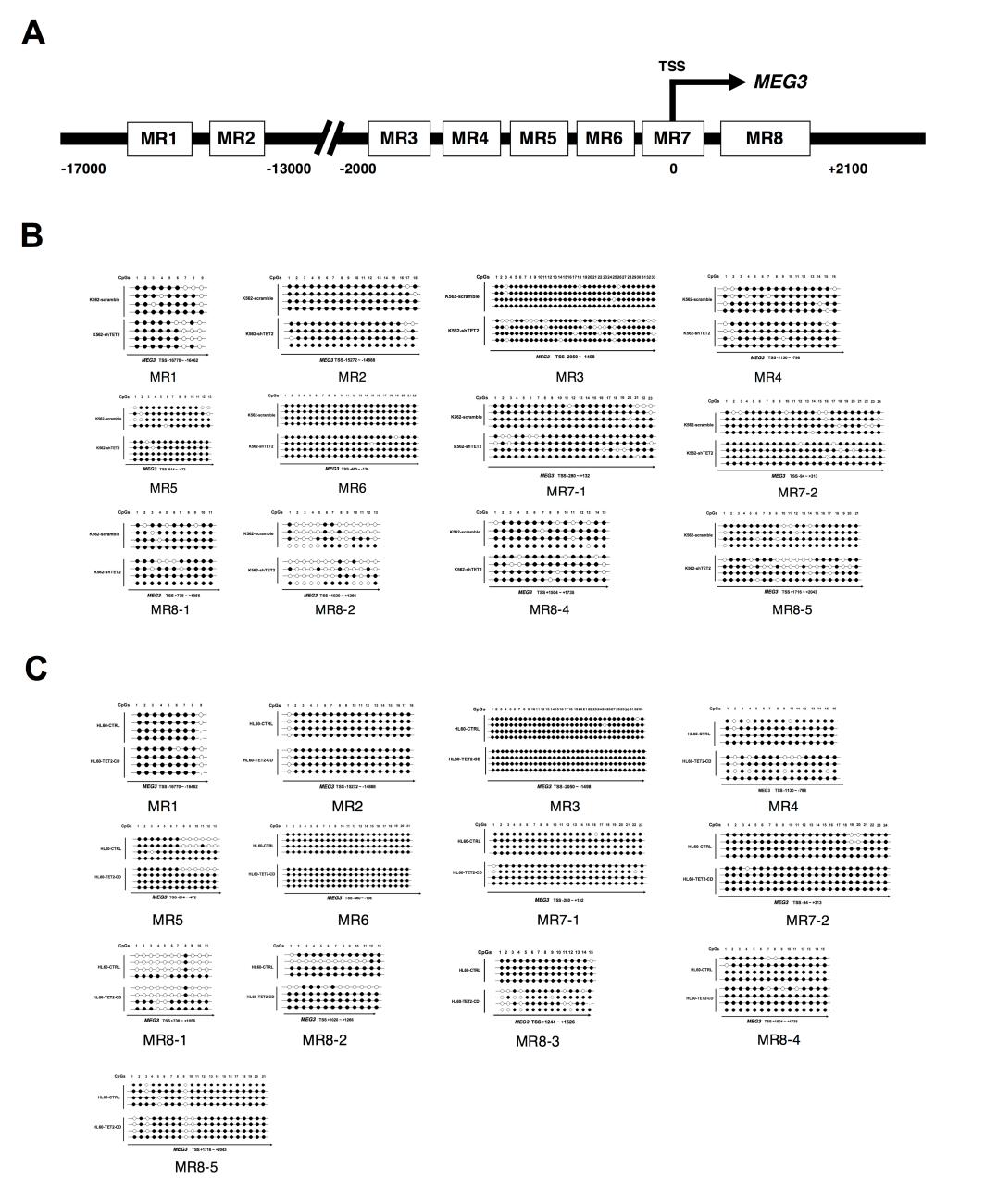


**Supplementary Figure 7.** (A) NB4 and 293T cell lines were transfected with a *WT1* construct along with Flag-TET2^CD^ construct. IP was carried out with either an anti-Flag antibody, an anti-WT1 antibody or an equivalent amount of IgG. (B, C) WT1 was transiently overexpressed either singly or with shRNA against *TET2* in U937 and NB4 cell lines, RT-qPCR was examined for the mRNA expression of *WT1* and *TET2*. (D) Reduction of *MEG3* promoter activity by shWT1-1 either singly or with Flag-TET2^CD^ in KG-1 cells. Shown were representative images of three independent experiments. **p* < 0.05; ***p* < 0.01; ****p* < 0.001; N.S, not significant.

**Supplementary Figure 7**

**
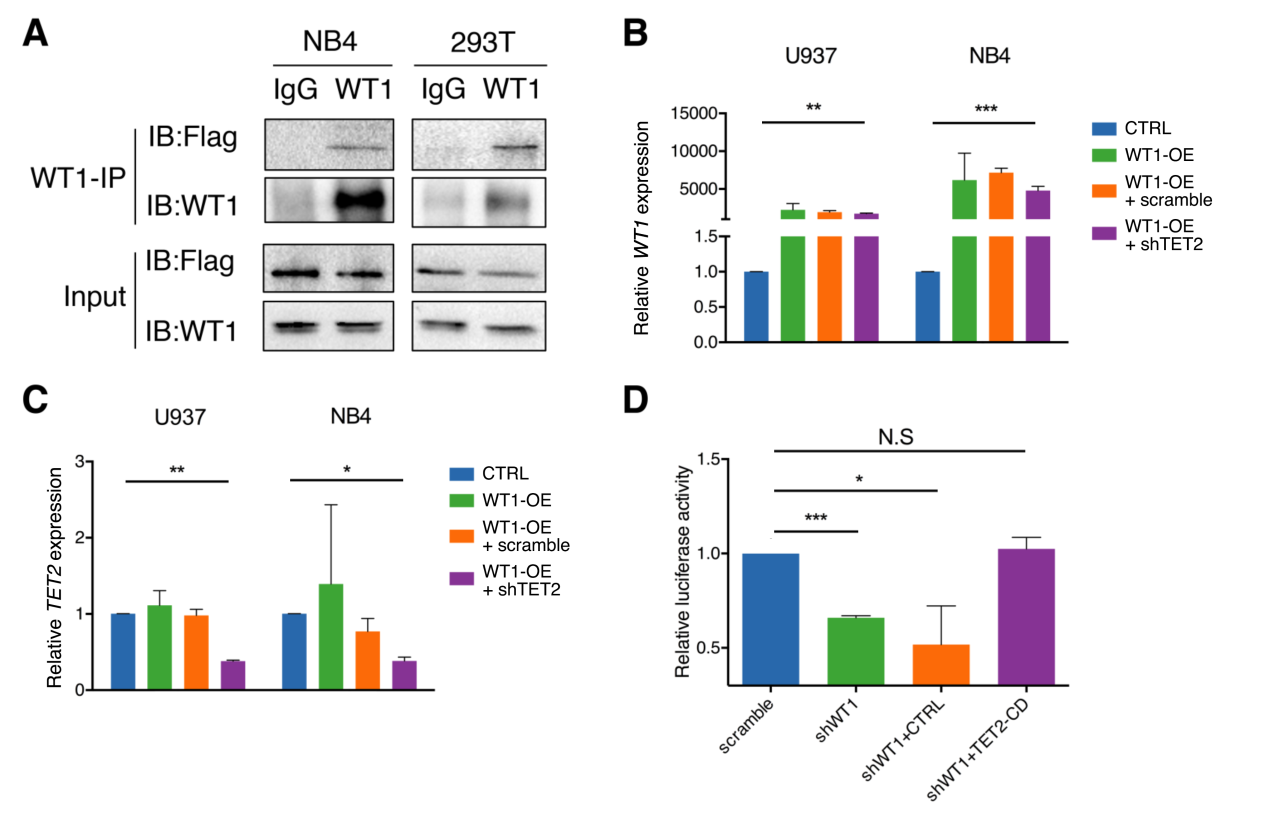
**

**Supplementary Tables**

**Supplementary Table 1-1. Mutations of *TET2*, *WT1* and *TP53* in CD34^+^ samples**

| Sample No. | #Chr | Start | End | Ref | Alt | Func.  refGene | Gene  refGene | ExonicFunc  refGene | AA Change  refGene | cytoBand | snp138 | cosmic77 | Mutation  ratio |
| --- | --- | --- | --- | --- | --- | --- | --- | --- | --- | --- | --- | --- | --- |
| 2 | . | . | . | . | . | . | . | . | . | . | . | . | . |
| 25 | chr4 | 106196951 | 106196951 | A | G | exonic | TET2 | nonsynonymous SNV | TET2:NM_001127208:exon11:c.A5284G:p.I1762V | 4q24 | rs2454206 | ID=COSM3760322;CNT=8;OCCURENCE=5(prostate),2(haematopoietic_and_lymphoid_tissue),1(thyroid) | 49.74% |
| 25 | chr4 | 106155185 | 106155185 | C | G | exonic | TET2 | nonsynonymous SNV | TET2:NM_001127208:exon3:c.C86G:p.P29R | 4q24 | rs12498609 | ID=COSM5020248,COSM5020249;CNT=2;OCCURENCE=2(soft_tissue) | 33.55% |
| 25 | chr17 | 7578506 | 7578506 | - | C | exonic | TP53 | frameshift insertion | TP53:NM_000546:exon5:c.423_424insG:p.P142fs | 17p13.1 | . | . | 5.63% |
| 28 | chr4 | 106196951 | 106196951 | A | G | exonic | TET2 | nonsynonymous SNV | TET2:NM_001127208:exon11:c.A5284G:p.I1762V | 4q24 | rs2454206 | ID=COSM3760322;CNT=8;OCCURENCE=5(prostate),2(haematopoietic_and_lymphoid_tissue),1(thyroid) | 50.07% |
| 39 | chr4 | 106196951 | 106196951 | A | G | exonic | TET2 | nonsynonymous SNV | TET2:NM_001127208:exon11:c.A5284G:p.I1762V | 4q24 | rs2454206 | ID=COSM3760322;CNT=8;OCCURENCE=5(prostate),2(haematopoietic_and_lymphoid_tissue),1(thyroid) | 99.56% |
| 40 | chr4 | 106157703 | 106157703 | T | G | exonic | TET2 | nonsynonymous SNV | TET2:NM_001127208:exon3:c.T2604G:p.F868L | 4q24 | rs147836249 | ID=COSM87107;CNT=7;OCCURENCE=7(haematopoietic_and_lymphoid_tissue) | 37.20% |
| 40 | chr4 | 106196951 | 106196951 | A | G | exonic | TET2 | nonsynonymous SNV | TET2:NM_001127208:exon11:c.A5284G:p.I1762V | 4q24 | rs2454206 | ID=COSM3760322;CNT=8;OCCURENCE=5(prostate),2(haematopoietic_and_lymphoid_tissue),1(thyroid) | 49.46% |
| 40 | chr4 | 106155185 | 106155185 | C | G | exonic | TET2 | nonsynonymous SNV | TET2:NM_001127208:exon3:c.C86G:p.P29R | 4q24 | rs12498609 | ID=COSM5020248,COSM5020249;CNT=2;OCCURENCE=2(soft_tissue) | 43.14% |
| 40 | chr4 | 106157703 | 106157703 | T | G | exonic | TET2 | nonsynonymous SNV | TET2:NM_001127208:exon3:c.T2604G:p.F868L | 4q24 | rs147836249 | ID=COSM87107;CNT=7;OCCURENCE=7(haematopoietic_and_lymphoid_tissue) | 33.13% |
| 41 | chr4 | 106196951 | 106196951 | A | G | exonic | TET2 | nonsynonymous SNV | TET2:NM_001127208:exon11:c.A5284G:p.I1762V | 4q24 | rs2454206 | ID=COSM3760322;CNT=8;OCCURENCE=5(prostate),2(haematopoietic_and_lymphoid_tissue),1(thyroid) | 52.20% |
| 42 | . | . | . | . | . | . | . | . | . | . | . | . | . |
| 43 | chr4 | 106155185 | 106155185 | C | G | exonic | TET2 | nonsynonymous SNV | TET2:NM_001127208:exon3:c.C86G:p.P29R | 4q24 | rs12498609 | ID=COSM5020248,COSM5020249;CNT=2;OCCURENCE=2(soft_tissue) | 49.92% |
| 44 | chr4 | 106196951 | 106196951 | A | G | exonic | TET2 | nonsynonymous SNV | TET2:NM_001127208:exon11:c.A5284G:p.I1762V | 4q24 | rs2454206 | ID=COSM3760322;CNT=8;OCCURENCE=5(prostate),2(haematopoietic_and_lymphoid_tissue),1(thyroid) | 50.85% |
| 44 | chr4 | 106155751 | 106155751 | G | A | exonic | TET2 | nonsynonymous SNV | TET2:NM_001127208:exon3:c.G652A:p.V218M | 4q24 | rs6843141 | ID=COSM4416139,COSM4416140;CNT=1;OCCURENCE=1(haematopoietic_and_lymphoid_tissue) | 47.60% |
| 44 | chr17 | 7578523 | 7578523 | - | G | exonic | TP53 | frameshift insertion | TP53:NM_000546:exon5:c.406dupC:p.Q136fs | 17p13.1 | . | . | 5.55% |
| 45 | chr4 | 106155185 | 106155185 | C | G | exonic | TET2 | nonsynonymous SNV | TET2:NM_001127208:exon3:c.C86G:p.P29R | 4q24 | rs12498609 | ID=COSM5020248,COSM5020249;CNT=2;OCCURENCE=2(soft_tissue) | 47.93% |
| 47 | . | . | . | . | . | . | . | . | . | . | . | . | . |
| 49 | chr4 | 106155185 | 106155185 | C | G | exonic | TET2 | nonsynonymous SNV | TET2:NM_001127208:exon3:c.C86G:p.P29R | 4q24 | rs12498609 | ID=COSM5020248,COSM5020249;CNT=2;OCCURENCE=2(soft_tissue) | 99.39% |
| 55 | chr4 | 106196829 | 106196829 | T | G | exonic | TET2 | nonsynonymous SNV | TET2:NM_001127208:exon11:c.T5162G:p.L1721W | 4q24 | rs34402524 | ID=COSM5020013;CNT=4;OCCURENCE=4(soft_tissue) | 46.04% |
| 56 | chr4 | 106155185 | 106155185 | C | G | exonic | TET2 | nonsynonymous SNV | TET2:NM_001127208:exon3:c.C86G:p.P29R | 4q24 | rs12498609 | ID=COSM5020248,COSM5020249;CNT=2;OCCURENCE=2(soft_tissue) | 49.19% |
| 57 | . | . | . | . | . | . | . | . | . | . | . | . | . |

**Supplementary Table 1-2. Mutations of *TET2*, *WT1* and *TP53* in AML samples**

| Sample No. | #Chr | Start | End | Ref | Alt | Func.  refGene | Gene  refGene | ExonicFunc  refGene | AA Change  refGene | cytoBand | snp138 | cosmic77 | Mutation  ratio |
| --- | --- | --- | --- | --- | --- | --- | --- | --- | --- | --- | --- | --- | --- |
| 1 | chr4 | 106155185 | 106155185 | C | G | exonic | TET2 | nonsynonymous SNV | TET2:NM_001127208:exon3:c.C86G:p.P29R | 4q24 | rs12498609 | ID=COSM5020248,COSM5020249;CNT=2;OCCURENCE=2(soft_tissue) | 99.59% |
| 3 | chr4 | 106155185 | 106155185 | C | G | exonic | TET2 | nonsynonymous SNV | TET2:NM_001127208:exon3:c.C86G:p.P29R | 4q24 | rs12498609 | ID=COSM5020248,COSM5020249;CNT=2;OCCURENCE=2(soft_tissue) | 52.78% |
| 4 | chr4 | 106196951 | 106196951 | A | G | exonic | TET2 | nonsynonymous SNV | TET2:NM_001127208:exon11:c.A5284G:p.I1762V | 4q24 | rs2454206 | ID=COSM3760322;CNT=8;OCCURENCE=5(prostate),2(haematopoietic_and_lymphoid_tissue),1(thyroid) | 50.83% |
| 5 | chr4 | 106196951 | 106196951 | A | G | exonic | TET2 | nonsynonymous SNV | TET2:NM_001127208:exon11:c.A5284G:p.I1762V | 4q24 | rs2454206 | ID=COSM3760322;CNT=8;OCCURENCE=5(prostate),2(haematopoietic_and_lymphoid_tissue),1(thyroid) | 48.88% |
| 6 | chr4 | 106155487 | 106155487 | G | T | exonic | TET2 | stopgain | TET2:NM_001127208:exon3:c.G388T:p.E130X | 4q24 | . | . | 33.47% |
| 7 | chr4 | 106155185 | 106155185 | C | G | exonic | TET2 | nonsynonymous SNV | TET2:NM_001127208:exon3:c.C86G:p.P29R | 4q24 | rs12498609 | ID=COSM5020248,COSM5020249;CNT=2;OCCURENCE=2(soft_tissue) | 49.83% |
| 7 | chr4 | 106155751 | 106155751 | G | A | exonic | TET2 | nonsynonymous SNV | TET2:NM_001127208:exon3:c.G652A:p.V218M | 4q24 | rs6843141 | ID=COSM4416139,COSM4416140;CNT=1;OCCURENCE=1(haematopoietic_and_lymphoid_tissue) | 45.91% |
| 8 | chr4 | 106155185 | 106155185 | C | G | exonic | TET2 | nonsynonymous SNV | TET2:NM_001127208:exon3:c.C86G:p.P29R | 4q24 | rs12498609 | ID=COSM5020248,COSM5020249;CNT=2;OCCURENCE=2(soft_tissue) | 51.82% |
| 8 | chr4 | 106190797 | 106190797 | C | A | exonic | TET2 | nonsynonymous SNV | TET2:NM_001127208:exon9:c.C4075A:p.R1359S | 4q24 | . | . | 50.40% |
| 8 | chr4 | 106157446 | 106157449 | GAAT | - | exonic | TET2 | frameshift deletion | TET2:NM_001127208:exon3:c.2347_2350del:p.E783fs | 4q24 | . | . | 42.45% |
| 9 | chr4 | 106197000 | 106197000 | A | G | exonic | TET2 | nonsynonymous SNV | TET2:NM_001127208:exon11:c.A5333G:p.H1778R | 4q24 | rs62621450 | . | 92.63% |
| 9 | chr4 | 106157797 | 106157797 | A | T | exonic | TET2 | stopgain | TET2:NM_001127208:exon3:c.A2698T:p.K900X | 4q24 | . | . | 88.66% |
| 10 | chr4 | 106155185 | 106155185 | C | G | exonic | TET2 | nonsynonymous SNV | TET2:NM_001127208:exon3:c.C86G:p.P29R | 4q24 | rs12498609 | ID=COSM5020248,COSM5020249;CNT=2;OCCURENCE=2(soft_tissue) | 99.87% |
| 10 | chr4 | 106197357 | 106197357 | T | A | exonic | TET2 | nonsynonymous SNV | TET2:NM_001127208:exon11:c.T5690A:p.I1897N | 4q24 | . | . | 47.36% |
| 10 | chr4 | 106158407 | 106158407 | - | TT | exonic | TET2 | frameshift insertion | TET2:NM_001127208:exon3:c.3308_3309insTT:p.N1103fs | 4q24 | . | . | 43.66% |
| 11 | chr4 | 106155185 | 106155185 | C | G | exonic | TET2 | nonsynonymous SNV | TET2:NM_001127208:exon3:c.C86G:p.P29R | 4q24 | rs12498609 | ID=COSM5020248,COSM5020249;CNT=2;OCCURENCE=2(soft_tissue) | 63.06% |
| 11 | chr4 | 106156625 | 106156625 | C | G | exonic | TET2 | stopgain | TET2:NM_001127208:exon3:c.C1526G:p.S509X | 4q24 | . | ID=COSM41736,COSM1133708;CNT=5;OCCURENCE=4(haematopoietic_and_lymphoid_tissue),1(urinary_tract) | 17.38% |
| 11 | chr4 | 106157856 | 106157856 | - | T | exonic | TET2 | frameshift insertion | TET2:NM_001127208:exon3:c.2758dupT:p.Y919fs | 4q24 | . | . | 10.61% |
| 11 | chr4 | 106196213 | 106196213 | C | T | exonic | TET2 | stopgain | TET2:NM_001127208:exon11:c.C4546T:p.R1516X | 4q24 | rs370735654 | ID=COSM43420;CNT=11;OCCURENCE=11(haematopoietic_and_lymphoid_tissue) | 3.37% |
| 12 | chr4 | 106155185 | 106155185 | C | G | exonic | TET2 | nonsynonymous SNV | TET2:NM_001127208:exon3:c.C86G:p.P29R | 4q24 | rs12498609 | ID=COSM5020248,COSM5020249;CNT=2;OCCURENCE=2(soft_tissue) | 51.41% |
| 13 | chr17 | 7578236 | 7578236 | A | C | exonic | TP53 | nonsynonymous SNV | TP53:NM_000546:exon6:c.T613G:p.Y205D | 17p13.1 | . | ID=COSM4946199,COSM1564190,COSM1564188,COSM1564191,COSM1564189,COSM43844,COSM4946198;CNT=34;OCCURENCE=6(upper_aerodigestive_tract),6(large_intestine),4(skin),4(breast),4(pancreas),2(prostate),2(oesophagus),2(central_nervous_system),2(stomach),2(urinary_tract) | 86.96% |
| 13 | chr4 | 106155185 | 106155185 | C | G | exonic | TET2 | nonsynonymous SNV | TET2:NM_001127208:exon3:c.C86G:p.P29R | 4q24 | rs12498609 | ID=COSM5020248,COSM5020249;CNT=2;OCCURENCE=2(soft_tissue) | 51.40% |
| 14 | chr4 | 106158215 | 106158215 | C | T | exonic | TET2 | nonsynonymous SNV | TET2:NM_001127208:exon3:c.C3116T:p.S1039L | 4q24 | rs111678678 | . | 53.38% |
| 14 | chr4 | 106155751 | 106155751 | G | A | exonic | TET2 | nonsynonymous SNV | TET2:NM_001127208:exon3:c.G652A:p.V218M | 4q24 | rs6843141 | ID=COSM4416139,COSM4416140;CNT=1;OCCURENCE=1(haematopoietic_and_lymphoid_tissue) | 50.60% |
| 15 | . | . | . | . | . | . | . | . | . | . | . | . | . |
| 16 | chr4 | 106196951 | 106196951 | A | G | exonic | TET2 | nonsynonymous SNV | TET2:NM_001127208:exon11:c.A5284G:p.I1762V | 4q24 | rs2454206 | ID=COSM3760322;CNT=8;OCCURENCE=5(prostate),2(haematopoietic_and_lymphoid_tissue),1(thyroid) | 48.54% |
| 16 | chr11 | 32417907 | 32417919 | GCCGACCGTACAA | - | exonic | WT1 | frameshift deletion | WT1:NM_000378:exon6:c.1082_1094del:p.L361fs | 11p13 | . | . | 1.09% |
| 17 | . | . | . | . | . | . | . | . | . | . | . | . | . |
| 18 | chr4 | 106156041 | 106156041 | T | - | exonic | TET2 | frameshift deletion | TET2:NM_001127208:exon3:c.942delT:p.C314fs | 4q24 | . | . | 5.59% |
| 19 | chr4 | 106190797 | 106190797 | C | T | exonic | TET2 | nonsynonymous SNV | TET2:NM_001127208:exon9:c.C4075T:p.R1359C | 4q24 | . | ID=COSM41649;CNT=6;OCCURENCE=5(haematopoietic_and_lymphoid_tissue),1(cervix) | 44.55% |
| 19 | chr4 | 106180830 | 106180832 | TTT | - | exonic | TET2 | nonframeshift deletion | TET2:NM_001127208:exon7:c.3858_3860del:p.1286_1287del | 4q24 | . | . | 32.28% |
| 20 | chr4 | 106155185 | 106155185 | C | G | exonic | TET2 | nonsynonymous SNV | TET2:NM_001127208:exon3:c.C86G:p.P29R | 4q24 | rs12498609 | ID=COSM5020248,COSM5020249;CNT=2;OCCURENCE=2(soft_tissue) | 52.87% |
| 20 | chr4 | 106196951 | 106196951 | A | G | exonic | TET2 | nonsynonymous SNV | TET2:NM_001127208:exon11:c.A5284G:p.I1762V | 4q24 | rs2454206 | ID=COSM3760322;CNT=8;OCCURENCE=5(prostate),2(haematopoietic_and_lymphoid_tissue),1(thyroid) | 46.03% |
| 21 | chr4 | 106155185 | 106155185 | C | G | exonic | TET2 | nonsynonymous SNV | TET2:NM_001127208:exon3:c.C86G:p.P29R | 4q24 | rs12498609 | ID=COSM5020248,COSM5020249;CNT=2;OCCURENCE=2(soft_tissue) | 99.55% |
| 21 | chr11 | 32417907 | 32417907 | - | CCGA | exonic | WT1 | frameshift insertion | WT1:NM_000378:exon6:c.1093_1094insTCGG:p.A365fs,WT1:NM_001198552:exon6:c.457_458insTCGG:p.A153fs,WT1:NM_001198551:exon7:c.508_509insTCGG:p.A170fs,WT1:NM_024424:exon7:c.1144_1145insTCGG:p.A382fs,WT1:NM_024426:exon7:c.1144_1145insTCGG:p.A382fs | 11p13 | . | ID=COSM21392,COSM1166613,COSM5487332;CNT=46;OCCURENCE=46(haematopoietic_and_lymphoid_tissue) | 46.44% |
| 22 | chr4 | 106196951 | 106196951 | A | G | exonic | TET2 | nonsynonymous SNV | TET2:NM_001127208:exon11:c.A5284G:p.I1762V | 4q24 | rs2454206 | ID=COSM3760322;CNT=8;OCCURENCE=5(prostate),2(haematopoietic_and_lymphoid_tissue),1(thyroid) | 48.12% |
| 23 | chr4 | 106196951 | 106196951 | A | G | exonic | TET2 | nonsynonymous SNV | TET2:NM_001127208:exon11:c.A5284G:p.I1762V | 4q24 | rs2454206 | ID=COSM3760322;CNT=8;OCCURENCE=5(prostate),2(haematopoietic_and_lymphoid_tissue),1(thyroid) | 49.15% |
| 23 | chr4 | 106196829 | 106196829 | T | G | exonic | TET2 | nonsynonymous SNV | TET2:NM_001127208:exon11:c.T5162G:p.L1721W | 4q24 | rs34402524 | ID=COSM5020013;CNT=4;OCCURENCE=4(soft_tissue) | 39.75% |
| 24 | chr4 | 106196951 | 106196951 | A | G | exonic | TET2 | nonsynonymous SNV | TET2:NM_001127208:exon11:c.A5284G:p.I1762V | 4q24 | rs2454206 | ID=COSM3760322;CNT=8;OCCURENCE=5(prostate),2(haematopoietic_and_lymphoid_tissue),1(thyroid) | 51.17% |
| 26 | chr4 | 106155185 | 106155185 | C | G | exonic | TET2 | nonsynonymous SNV | TET2:NM_001127208:exon3:c.C86G:p.P29R,TET2:NM_017628:exon3:c.C86G:p.P29R | 4q24 | rs12498609 | ID=COSM5020248,COSM5020249;CNT=2;OCCURENCE=2(soft_tissue) | 42.94% |
| 27 | chr4 | 106155185 | 106155185 | C | G | exonic | TET2 | nonsynonymous SNV | TET2:NM_001127208:exon3:c.C86G:p.P29R | 4q24 | rs12498609 | ID=COSM5020248,COSM5020249;CNT=2;OCCURENCE=2(soft_tissue) | 99.72% |
| 27 | chr4 | 106180790 | 106180790 | - | CC | exonic | TET2 | frameshift insertion | TET2:NM_001127208:exon7:c.3818_3819insCC:p.C1273fs | 4q24 | . | . | 1.10% |
| 29 | chr4 | 106155185 | 106155185 | C | G | exonic | TET2 | nonsynonymous SNV | TET2:NM_001127208:exon3:c.C86G:p.P29R | 4q24 | rs12498609 | ID=COSM5020248,COSM5020249;CNT=2;OCCURENCE=2(soft_tissue) | 51.57% |
| 30 | chr4 | 106155185 | 106155185 | C | G | exonic | TET2 | nonsynonymous SNV | TET2:NM_001127208:exon3:c.C86G:p.P29R | 4q24 | rs12498609 | ID=COSM5020248,COSM5020249;CNT=2;OCCURENCE=2(soft_tissue) | 49.45% |
| 30 | chr4 | 106196951 | 106196951 | A | G | exonic | TET2 | nonsynonymous SNV | TET2:NM_001127208:exon11:c.A5284G:p.I1762V | 4q24 | rs2454206 | ID=COSM3760322;CNT=8;OCCURENCE=5(prostate),2(haematopoietic_and_lymphoid_tissue),1(thyroid) | 49.28% |
| 31 | chr4 | 106196829 | 106196829 | T | G | exonic | TET2 | nonsynonymous SNV | TET2:NM_001127208:exon11:c.T5162G:p.L1721W | 4q24 | rs34402524 | ID=COSM5020013;CNT=4;OCCURENCE=4(soft_tissue) | 37.95% |
| 32 | chr4 | 106157002 | 106157002 | C | T | exonic | TET2 | stopgain | TET2:NM_001127208:exon3:c.C1903T:p.Q635X | 4q24 | . | ID=COSM46414;CNT=1;OCCURENCE=1(haematopoietic_and_lymphoid_tissue) | 47.72% |
| 32 | chr4 | 106196829 | 106196829 | T | G | exonic | TET2 | nonsynonymous SNV | TET2:NM_001127208:exon11:c.T5162G:p.L1721W | 4q24 | rs34402524 | ID=COSM5020013;CNT=4;OCCURENCE=4(soft_tissue) | 47.48% |
| 32 | chr4 | 106155185 | 106155185 | C | G | exonic | TET2 | nonsynonymous SNV | TET2:NM_001127208:exon3:c.C86G:p.P29R | 4q24 | rs12498609 | ID=COSM5020248,COSM5020249;CNT=2;OCCURENCE=2(soft_tissue) | 42.96% |
| 32 | chr4 | 106180785 | 106180785 | C | G | exonic | TET2 | nonsynonymous SNV | TET2:NM_001127208:exon7:c.C3813G:p.C1271W | 4q24 | . | ID=COSM120176;CNT=5;OCCURENCE=4(haematopoietic_and_lymphoid_tissue),1(bone) | 18.08% |
| 33 | chr4 | 106196951 | 106196951 | A | G | exonic | TET2 | nonsynonymous SNV | TET2:NM_001127208:exon11:c.A5284G:p.I1762V | 4q24 | rs2454206 | ID=COSM3760322;CNT=8;OCCURENCE=5(prostate),2(haematopoietic_and_lymphoid_tissue),1(thyroid) | 45.68% |
| 34 | chr4 | 106155185 | 106155185 | C | G | exonic | TET2 | nonsynonymous SNV | TET2:NM_001127208:exon3:c.C86G:p.P29R | 4q24 | rs12498609 | ID=COSM5020248,COSM5020249;CNT=2;OCCURENCE=2(soft_tissue) | 51.65% |
| 35 | chr4 | 106155185 | 106155185 | C | G | exonic | TET2 | nonsynonymous SNV | TET2:NM_001127208:exon3:c.C86G:p.P29R | 4q24 | rs12498609 | ID=COSM5020248,COSM5020249;CNT=2;OCCURENCE=2(soft_tissue) | 49.62% |
| 35 | chr4 | 106196951 | 106196951 | A | G | exonic | TET2 | nonsynonymous SNV | TET2:NM_001127208:exon11:c.A5284G:p.I1762V | 4q24 | rs2454206 | ID=COSM3760322;CNT=8;OCCURENCE=5(prostate),2(haematopoietic_and_lymphoid_tissue),1(thyroid) | 49.48% |
| 36 | chr4 | 106196951 | 106196951 | A | G | exonic | TET2 | nonsynonymous SNV | TET2:NM_001127208:exon11:c.A5284G:p.I1762V | 4q24 | rs2454206 | ID=COSM3760322;CNT=8;OCCURENCE=5(prostate),2(haematopoietic_and_lymphoid_tissue),1(thyroid) | 50.17% |
| 36 | chr4 | 106155751 | 106155751 | G | A | exonic | TET2 | nonsynonymous SNV | TET2:NM_001127208:exon3:c.G652A:p.V218M | 4q24 | rs6843141 | ID=COSM4416139,COSM4416140;CNT=1;OCCURENCE=1(haematopoietic_and_lymphoid_tissue) | 47.05% |
| 37 | chr4 | 106155185 | 106155185 | C | G | exonic | TET2 | nonsynonymous SNV | TET2:NM_001127208:exon3:c.C86G:p.P29R | 4q24 | rs12498609 | ID=COSM5020248,COSM5020249;CNT=2;OCCURENCE=2(soft_tissue) | 47.93% |
| 38 | chr4 | 106196951 | 106196951 | A | G | exonic | TET2 | nonsynonymous SNV | TET2:NM_001127208:exon11:c.A5284G:p.I1762V | 4q24 | rs2454206 | ID=COSM3760322;CNT=8;OCCURENCE=5(prostate),2(haematopoietic_and_lymphoid_tissue),1(thyroid) | 47.51% |
| 46 | chr4 | 106155778 | 106155778 | - | A | exonic | TET2 | frameshift insertion | TET2:NM_001127208:exon3:c.680dupA:p.E227fs | 4q24 | . | ID=COSM2952696,COSM2952695;CNT=8;OCCURENCE=4(large_intestine),4(haematopoietic_and_lymphoid_tissue) | 46.30% |
| 48 | chr4 | 106155185 | 106155185 | C | G | exonic | TET2 | nonsynonymous SNV | TET2:NM_001127208:exon3:c.C86G:p.P29R | 4q24 | rs12498609 | ID=COSM5020248,COSM5020249;CNT=2;OCCURENCE=2(soft_tissue) | 51.05% |
| 48 | chr4 | 106155738 | 106155738 | - | A | exonic | TET2 | frameshift insertion | TET2:NM_001127208:exon3:c.639_640insA:p.V213fs | 4q24 | . | . | 1.16% |
| 50 | chr4 | 106155185 | 106155185 | C | G | exonic | TET2 | nonsynonymous SNV | TET2:NM_001127208:exon3:c.C86G:p.P29R | 4q24 | rs12498609 | ID=COSM5020248,COSM5020249;CNT=2;OCCURENCE=2(soft_tissue) | 80.46% |
| 50 | chr17 | 7578400 | 7578400 | G | C | exonic | TP53 | nonsynonymous SNV | TP53:NM_000546:exon5:c.C530G:p.P177R | 17p13.1 | . | ID=COSM1640849,COSM117224,COSM10651,COSM117223,COSM4947779,COSM117222,COSM117221;CNT=18;OCCURENCE=4(stomach),3(central_nervous_system),2(breast),2(pancreas),2(large_intestine),1(prostate),1(upper_aerodigestive_tract),1(liver),1(lung),1(ovary) | 41.08% |
| 50 | chr4 | 106155751 | 106155751 | G | A | exonic | TET2 | nonsynonymous SNV | TET2:NM_001127208:exon3:c.G652A:p.V218M | 4q24 | rs6843141 | ID=COSM4416139,COSM4416140;CNT=1;OCCURENCE=1(haematopoietic_and_lymphoid_tissue) | 18.39% |
| 51 | . | . | . | . | . | . | . | . | . | . | . | . | . |
| 52 | chr4 | 106155185 | 106155185 | C | G | exonic | TET2 | nonsynonymous SNV | TET2:NM_001127208:exon3:c.C86G:p.P29R | 4q24 | rs12498609 | ID=COSM5020248,COSM5020249;CNT=2;OCCURENCE=2(soft_tissue) | 53.28% |
| 53 | chr4 | 106155881 | 106155881 | C | - | exonic | TET2 | frameshift deletion | TET2:NM_001127208:exon3:c.782delC:p.S261fs | 4q24 | . | . | 48.53% |
| 53 | chr4 | 106180783 | 106180783 | - | G | exonic | TET2 | frameshift insertion | TET2:NM_001127208:exon7:c.3812dupG:p.C1271fs | 4q24 | . | ID=COSM4170106;CNT=1;OCCURENCE=1(haematopoietic_and_lymphoid_tissue) | 47.56% |
| 54 | chr4 | 106155185 | 106155185 | C | G | exonic | TET2 | nonsynonymous SNV | TET2:NM_001127208:exon3:c.C86G:p.P29R | 4q24 | rs12498609 | ID=COSM5020248,COSM5020249;CNT=2;OCCURENCE=2(soft_tissue) | 49.18% |

**Supplementary Table 1-3. Mutations of *TET2*, *WT1* and *TP53* in AML cell lines**

| Cell lines | #Chr | Start | End | Ref | Alt | Func.  refGene | Gene  refGene | ExonicFunc  refGene | AA Change  refGene | cytoBand | snp138 | cosmic77 | Mutation  ratio |
| --- | --- | --- | --- | --- | --- | --- | --- | --- | --- | --- | --- | --- | --- |
| MOLM-13 | chr4 | 106155751 | 106155751 | G | A | exonic | TET2 | nonsynonymous SNV | TET2:NM_001127208:exon3:c.G652A:p.V218M | 4q24 | rs6843141 | ID=COSM4416139,COSM4416140;CNT=1;OCCURENCE=1(haematopoietic_and_lymphoid_tissue) | 53% |
| MOLM-13 | chr4 | 106196951 | 106196951 | A | G | exonic | TET2 | nonsynonymous SNV | TET2:NM_001127208:exon11:c.A5284G:p.I1762V | 4q24 | rs2454206 | ID=COSM3760322;CNT=8;OCCURENCE=5(prostate),2(haematopoietic_and_lymphoid_tissue),1(thyroid) | 53% |
| Kasumi-1 | chr17 | 7577538 | 7577538 | C | T | exonic | TP53 | nonsynonymous SNV | TP53:NM_000546:exon7:c.G743A:p.R248Q | 17p13.1 | rs11540652 | ID=COSM1640830,COSM10662,COSM99021,COSM99020,COSM3356964,COSM99602;CNT=1474;OCCURENCE=338(large_intestine),156(breast),138(haematopoietic_and_lymphoid_tissue),108(central_nervous_system),94(oesophagus),94(stomach),88(upper_aerodigestive_tract),74(urinary_tract),72(ovary),64(lung),36(pancreas),36(endometrium),34(prostate),30(skin),18(liver),16(vulva),14(small_intestine),14(biliary_tract),12(bone),10(thyroid),8(kidney),8(cervix),2(NS),2(pituitary),2(pleura),2(adrenal_gland),2(peritoneum),2(soft_tissue) | 99% |
| Kasumi-1 | chr4 | 106196951 | 106196951 | A | G | exonic | TET2 | nonsynonymous SNV | TET2:NM_001127208:exon11:c.A5284G:p.I1762V | 4q24 | rs2454206 | ID=COSM3760322;CNT=8;OCCURENCE=5(prostate),2(haematopoietic_and_lymphoid_tissue),1(thyroid) | 51% |
| U937 | chr11 | 32417947 | 32417947 | G | A | exonic | WT1 | stopgain | WT1:NM_000378:exon6:c.C1054T:p.R352X,WT1:NM_001198552:exon6:c.C418T:p.R140X,WT1:NM_001198551:exon7:c.C469T:p.R157X,WT1:NM_024424:exon7:c.C1105T:p.R369X,WT1:NM_024426:exon7:c.C1105T:p.R369X | 11p13 | . | ID=COSM21441,COSM2114546,COSM4191076;CNT=6;OCCURENCE=3(haematopoietic_and_lymphoid_tissue),2(kidney),1(pancreas) | 49% |
| HL-60 | . | . | . | . | . |  | . | . | . | . | . | . | . |
| KG-1 | chr4 | 106155751 | 106155751 | G | A | exonic | TET2 | nonsynonymous SNV | TET2:NM_001127208:exon3:c.G652A:p.V218M | 4q24 | rs6843141 | ID=COSM4416139,COSM4416140;CNT=1;OCCURENCE=1(haematopoietic_and_lymphoid_tissue) | 43% |
| K562 | chr17 | 7578523 | 7578523 | - | G | exonic | TP53 | frameshift insertion | TP53:NM_000546:exon5:c.406dupC:p.Q136fs | 17p13.1 | . | . | 96% |
| K562 | chr4 | 106196829 | 106196829 | T | G | exonic | TET2 | nonsynonymous SNV | TET2:NM_001127208:exon11:c.T5162G:p.L1721W | 4q24 | rs34402524 | ID=COSM5020013;CNT=4;OCCURENCE=4(soft_tissue) | 65% |
| K562 | chr4 | 106196951 | 106196951 | A | G | exonic | TET2 | nonsynonymous SNV | TET2:NM_001127208:exon11:c.A5284G:p.I1762V | 4q24 | rs2454206 | ID=COSM3760322;CNT=8;OCCURENCE=5(prostate),2(haematopoietic_and_lymphoid_tissue),1(thyroid) | 34% |
| TF-1 | chr17 | 7577529 | 7577529 | A | - | exonic | TP53 | frameshift deletion | TP53:NM_000546:exon7:c.752delT:p.I251fs | 17p13.1 | . | ID=COSM45630;CNT=1;OCCURENCE=1(large_intestine) | 49% |
| TF-1 | chr4 | 106155751 | 106155751 | G | A | exonic | TET2 | nonsynonymous SNV | TET2:NM_001127208:exon3:c.G652A:p.V218M | 4q24 | rs6843141 | ID=COSM4416139,COSM4416140;CNT=1;OCCURENCE=1(haematopoietic_and_lymphoid_tissue) | 46% |
| NB4 | chr17 | 7577538 | 7577538 | C | T | exonic | TP53 | nonsynonymous SNV | TP53:NM_000546:exon7:c.G743A:p.R248Q | 17p13.1 | rs11540652 | ID=COSM1640830,COSM10662,COSM99021,COSM99020,COSM3356964,COSM99602;CNT=1474;OCCURENCE=338(large_intestine),156(breast),138(haematopoietic_and_lymphoid_tissue),108(central_nervous_system),94(oesophagus),94(stomach),88(upper_aerodigestive_tract),74(urinary_tract),72(ovary),64(lung),36(pancreas),36(endometrium),34(prostate),30(skin),18(liver),16(vulva),14(small_intestine),14(biliary_tract),12(bone),10(thyroid),8(kidney),8(cervix),2(NS),2(pituitary),2(pleura),2(adrenal_gland),2(peritoneum),2(soft_tissue) | 98% |

**Supplementary Table 2-1. Statistics on mutations of *TET2*, *WT1* and *TP53* in CD34^+^ samples**

| Sample No. | *TET2*-mut | *TET2*-  nonsynonymous SNV | *TET2*-  frameshift insertion | *TET2*-  frameshift deletion | *TET2*-stopgain | *WT1*-mut | *WT1*-  nonsynonymous SNV | *WT1*-  frameshift insertion | *WT1*-  frameshift deletion | *TP53*-mut | *TP53*-  nonsynonymous SNV | *TP53*-  frameshift insertion |
| --- | --- | --- | --- | --- | --- | --- | --- | --- | --- | --- | --- | --- |
| 2 | 0 | 0 | 0 | 0 | 0 | 0 | 0 | 0 | 0 | 0 | 0 | 0 |
| 25 | 2 | 2 | 0 | 0 | 0 | 0 | 0 | 0 | 0 | 1 | 0 | 1 |
| 28 | 1 | 1 | 0 | 0 | 0 | 0 | 0 | 0 | 0 | 0 | 0 | 0 |
| 39 | 2 | 2 | 0 | 0 | 0 | 0 | 0 | 0 | 0 | 0 | 0 | 0 |
| 40 | 3 | 3 | 0 | 0 | 0 | 0 | 0 | 0 | 0 | 0 | 0 | 0 |
| 41 | 1 | 1 | 0 | 0 | 0 | 0 | 0 | 0 | 0 | 0 | 0 | 0 |
| 42 | 0 | 0 | 0 | 0 | 0 | 0 | 0 | 0 | 0 | 0 | 0 | 0 |
| 43 | 1 | 1 | 0 | 0 | 0 | 0 | 0 | 0 | 0 | 0 | 0 | 0 |
| 44 | 2 | 2 | 0 | 0 | 0 | 0 | 0 | 0 | 0 | 1 | 0 | 1 |
| 45 | 1 | 1 | 0 | 0 | 0 | 0 | 0 | 0 | 0 | 0 | 0 | 0 |
| 47 | 0 | 0 | 0 | 0 | 0 | 0 | 0 | 0 | 0 | 0 | 0 | 0 |
| 49 | 1 | 1 | 0 | 0 | 0 | 0 | 0 | 0 | 0 | 0 | 0 | 0 |
| 55 | 1 | 1 | 0 | 0 | 0 | 0 | 0 | 0 | 0 | 0 | 0 | 0 |
| 56 | 1 | 1 | 0 | 0 | 0 | 0 | 0 | 0 | 0 | 0 | 0 | 0 |
| 57 | 0 | 0 | 0 | 0 | 0 | 0 | 0 | 0 | 0 | 0 | 0 | 0 |

**Supplementary Table 2-2. Statistics on mutations of *TET2*, *WT1* and *TP53* in AML samples**

| Sample No. | *TET2*-mut | *TET2*-  nonsynonymous SNV | *TET2*-  frameshift insertion | *TET2*-  frameshift deletion | *TET2*-stopgain | *WT1*-mut | *WT1*-  nonsynonymous SNV | *WT1*-  frameshift insertion | *WT1*-  frameshift deletion | *TP53*-mut | *TP53*-  nonsynonymous SNV | *TP53*-  frameshift insertion |
| --- | --- | --- | --- | --- | --- | --- | --- | --- | --- | --- | --- | --- |
| 6 | 1 | 0 | 0 | 0 | 1 | 0 | 0 | 0 | 0 | 0 | 0 | 0 |
| 8 | 3 | 2 | 0 | 1 | 0 | 0 | 0 | 0 | 0 | 0 | 0 | 0 |
| 9 | 2 | 1 | 0 | 0 | 1 | 0 | 0 | 0 | 0 | 0 | 0 | 0 |
| 10 | 3 | 2 | 1 | 0 | 0 | 0 | 0 | 0 | 0 | 0 | 0 | 0 |
| 11 | 4 | 1 | 1 | 0 | 2 | 0 | 0 | 0 | 0 | 0 | 0 | 0 |
| 16 | 1 | 1 | 0 | 0 | 0 | 1 | 0 | 0 | 1 | 0 | 0 | 0 |
| 18 | 1 | 1 | 0 | 1 | 0 | 0 | 0 | 0 | 0 | 0 | 0 | 0 |
| 19 | 2 | 1 | 0 | 1 | 0 | 0 | 0 | 0 | 0 | 0 | 0 | 0 |
| 21 | 1 | 1 | 0 | 0 | 0 | 1 | 0 | 1 | 0 | 0 | 0 | 0 |
| 27 | 2 | 1 | 1 | 0 | 0 | 0 | 0 | 0 | 0 | 0 | 0 | 0 |
| 32 | 4 | 3 | 0 | 0 | 1 | 0 | 0 | 0 | 0 | 0 | 0 | 0 |
| 46 | 0 | 0 | 1 | 0 | 0 | 0 | 0 | 0 | 0 | 0 | 0 | 0 |
| 48 | 0 | 1 | 1 | 0 | 0 | 0 | 0 | 0 | 0 | 0 | 0 | 0 |
| 53 | 2 | 0 | 1 | 1 | 0 | 0 | 0 | 0 | 0 | 0 | 0 | 0 |
| 1 | 1 | 1 | 0 | 0 | 0 | 0 | 0 | 0 | 0 | 0 | 0 | 0 |
| 3 | 1 | 1 | 0 | 0 | 0 | 0 | 0 | 0 | 0 | 0 | 0 | 0 |
| 4 | 1 | 1 | 0 | 0 | 0 | 0 | 0 | 0 | 0 | 0 | 0 | 0 |
| 5 | 1 | 1 | 0 | 0 | 0 | 0 | 0 | 0 | 0 | 0 | 0 | 0 |
| 7 | 2 | 2 | 0 | 0 | 0 | 0 | 0 | 0 | 0 | 0 | 0 | 0 |
| 12 | 1 | 1 | 0 | 0 | 0 | 0 | 0 | 0 | 0 | 0 | 0 | 0 |
| 13 | 1 | 1 | 0 | 0 | 0 | 0 | 0 | 0 | 0 | 1 | 1 | 0 |
| 14 | 2 | 2 | 0 | 0 | 0 | 0 | 0 | 0 | 0 | 0 | 0 | 0 |
| 15 | 0 | 0 | 0 | 0 | 0 | 0 | 0 | 0 | 0 | 0 | 0 | 0 |
| 17 | 0 | 0 | 0 | 0 | 0 | 0 | 0 | 0 | 0 | 0 | 0 | 0 |
| 20 | 2 | 2 | 0 | 0 | 0 | 0 | 0 | 0 | 0 | 0 | 0 | 0 |
| 22 | 1 | 1 | 0 | 0 | 0 | 0 | 0 | 0 | 0 | 0 | 0 | 0 |
| 23 | 2 | 2 | 0 | 0 | 0 | 0 | 0 | 0 | 0 | 0 | 0 | 0 |
| 24 | 1 | 1 | 0 | 0 | 0 | 0 | 0 | 0 | 0 | 0 | 0 | 0 |
| 26 | 1 | 1 | 0 | 0 | 0 | 0 | 0 | 0 | 0 | 0 | 0 | 0 |
| 29 | 1 | 1 | 0 | 0 | 0 | 0 | 0 | 0 | 0 | 0 | 0 | 0 |
| 30 | 2 | 2 | 0 | 0 | 0 | 0 | 0 | 0 | 0 | 0 | 0 | 0 |
| 31 | 1 | 1 | 0 | 0 | 0 | 0 | 0 | 0 | 0 | 0 | 0 | 0 |
| 33 | 1 | 1 | 0 | 0 | 0 | 0 | 0 | 0 | 0 | 0 | 0 | 0 |
| 34 | 1 | 1 | 0 | 0 | 0 | 0 | 0 | 0 | 0 | 0 | 0 | 0 |
| 35 | 2 | 2 | 0 | 0 | 0 | 0 | 0 | 0 | 0 | 0 | 0 | 0 |
| 36 | 2 | 2 | 0 | 0 | 0 | 0 | 0 | 0 | 0 | 0 | 0 | 0 |
| 37 | 1 | 1 | 0 | 0 | 0 | 0 | 0 | 0 | 0 | 0 | 0 | 0 |
| 38 | 1 | 1 | 0 | 0 | 0 | 0 | 0 | 0 | 0 | 0 | 0 | 0 |
| 50 | 0 | 2 | 0 | 0 | 0 | 0 | 0 | 0 | 0 | 1 | 1 | 0 |
| 51 | 0 | 0 | 0 | 0 | 0 | 0 | 0 | 0 | 0 | 0 | 0 | 0 |
| 52 | 1 | 1 | 0 | 0 | 0 | 0 | 0 | 0 | 0 | 0 | 0 | 0 |
| 54 | 1 | 1 | 0 | 0 | 0 | 0 | 0 | 0 | 0 | 0 | 0 | 0 |

**Supplementary Table 2-3. Statistics on mutations of *TET2*, *WT1* and *TP53* in AML cell lines**

| Cell lines | *TET2*-  mut | *TET2*-  nonsynonymous SNV | *TET2*-  frameshift insertion | *TET2*-  frameshift deletion | *TET2*-  stopgain | *WT1*-  mut | *WT1*-  nonsynonymous SNV | *WT1*-  frameshift insertion | *WT1*-  frameshift deletion | *WT1-*  *Stopgain* | *TP53*-  mut | *TP53*-  nonsynonymous SNV | *TP53*-  frameshift insertion |
| --- | --- | --- | --- | --- | --- | --- | --- | --- | --- | --- | --- | --- | --- |
| MOLM-13 | 2 | 2 | 0 | 0 | 0 | 0 | 0 | 0 | 0 | 0 | 0 | 0 | 0 |
| Kasumi-1 | 1 | 1 | 0 | 0 | 0 | 0 | 0 | 0 | 0 | 0 | 1 | 1 | 0 |
| U937 | 0 | 0 | 0 | 0 | 0 | 1 | 0 | 0 | 0 | 1 | 0 | 0 | 0 |
| HL-60 | 0 | 0 | 0 | 0 | 0 | 0 | 0 | 0 | 0 | 0 | 0 | 0 | 0 |
| KG-1 | 1 | 1 | 0 | 0 | 0 | 0 | 0 | 0 | 0 | 0 | 0 | 0 | 0 |
| K562 | 2 | 2 | 0 | 0 | 0 | 0 | 0 | 0 | 0 | 0 | 1 | 0 | 1 |
| TF-1 | 1 | 1 | 0 | 0 | 0 | 0 | 0 | 0 | 0 | 0 | 1 | 0 | 1 |
| NB4 | 0 | 0 | 0 | 0 | 0 | 0 | 0 | 0 | 0 | 0 | 1 | 1 | 0 |

**Supplementary Table 3. List of antibodies used in this study**

| Target | Source | Catalog No. |
| --- | --- | --- |
| WT1 | Proteintech | 12609-1-AP |
| MDM2 Antibody (SMP14) | Santa Cruz | sc-965 |
| p53 (FL-393) | Santa Cruz | sc-6243 |
| p-RB (Ser249/Thr252) | Santa Cruz | sc-16671 |
| RB (D20) Rabbit mAb | CST | 9313S |
| PCNA | CST | 2586 |
| AKT | CST | C67E7 |
| PI3K | abcam | ab191606 |
| DNMT3A | abcam | ab2850 |
| TET2 | abcam | ab94580 |
| BAX | abcam | ab32503 |
| NOXA | abcam | ab140129 |
| PUMA | abcam | ab33906 |
| Flag | Sigma | F1804 |
| CD45 | BD | 560976 |
| CD38 | BD | 561106 |
| ACTB | Bioss | bs-0061R |
| GAPDH | Bioss | bsm-0978M |

**Supplementary Table 4. List of oligonucleotides used in this study**

| Name | Sequence | Note |
| --- | --- | --- |
| shWT1-#1-Upper | 5'-CCGGGGAAACTTCAGTTGATCTTCTCTCGAGAGAAGATCAACTGAAGTTTCCTTTTTG-3' | shRNA cloning |
| shWT1-#1-Lower | 5'-AATTCAAAAAGGAAACTTCAGTTGATCTTCTCTCGAGAGAAGATCAACTGAAGTTTCC-3' | shRNA cloning |
| shWT1-#2-Upper | 5'-CCGGGGTGAATCTTGTCTAACATTCCTCGAGGAATGTTAGACAAGATTCACCTTTTTG-3' | shRNA cloning |
| shWT1-#2-Lower | 5'-AATTCAAAAAGGTGAATCTTGTCTAACATTCCTCGAGGAATGTTAGACAAGATTCACC-3' | shRNA cloning |
| ACTB-300bp-F | TCGTCCACCGCAAATGCTT | PCR |
| ACTB-300bp-R | ACTTCCTGTAACAACGCATC | PCR |
| MEG3-500bp-F | ACATACAAAGCAGCCACTCAC | PCR |
| MEG3-500bp-R | ACCTCCTCTATGCCAGATCCT | PCR |
| PGL4-MEG3  (-1248)-XhoI-F | CCGCTCGAGTTTTCAGCCCTGGAATCTCCC | PCR |
| PGL4-MEG3 (+143)-HindIII-R | CCCAAGCTTGATGCCGTCTTCCTTTTGC | PCR |
| ACTB-F | ATGTGGCCGAGGACTTTGATT | RT-qPCR |
| ACTB-R | AGTGGGGTGGCTTTTAGGATG | RT-qPCR |
| MEG3-F | GCCCTGACCTTTGCTATGCT | RT-qPCR |
| MEG3-R | TCGACAAAGACTGACACCCC | RT-qPCR |
| DNMT3A-F | ACGCAAAACAGAACCCAGT | RT-qPCR |
| DNMT3A-R | CATCTCGCTGTTTGAAAGCAC | RT-qPCR |
| TET2-F | AGGCTAGGCTGCTTTCGTAG | RT-qPCR |
| TET2-R | GAATGTTTGCCAGCCTCGTT | RT-qPCR |
| WT1-F | GACGCCCTACAGCAGTGACA | RT-qPCR |
| WT1-R | TGGTTATCGCTCTCGTACCCT | RT-qPCR |
| MEG3-F | TTTTCAGCCCTGGAATCTCCC | ChIP-qPCR |
| MEG3-R | AAATGTCCTCTCGCAAAGGTC | ChIP-qPCR |

**Supplementary Table 5. Bisulfite sequencing primers for *MEG3***

| Name | Primers | Tm (°C) | Product size (bp) |
| --- | --- | --- | --- |
| MR1 | ATTATTTTTTGGATAAGAGAGT | 56.8 | 384 |
|  | TTTAAAACCCTCAAAACGATT |  |  |
| MR2 | TTAGGTTGGAATTGTTAAGAGTTTGTGGATT | 53.3 | 385 |
|  | ATAAACTACACTACTAAAAACTACATTTAAA |  |  |
| MR3 | TTGATAGGAGAGATTGGATATTAGGTGTTTGG | 64.5 | 551 |
|  | CACCCCCTTTACAACCTATAAAACTTACCAAAAAC |  |  |
| MR4 | TGGGGTTTTTTGTTTATTTTTATTT | 59.3 | 333 |
|  | ATTAAAATAATCCCCACACACATAC |  |  |
| MR5 | TGTGTGGGGATTATTTTAATTTGGGGGTAG | 64.5 | 346 |
|  | TAAACCAAAAACTATCACCCCCTCCCAACC |  |  |
| MR6 | ATTTGGGAATTAGTTATGTCGTT | 59.1 | 325 |
|  | TAACCGCACCCAAATTACAAC |  |  |
| MR7-1 | TTGTGTTTGAATTTATTTTGTTTGG | 59.9 | 407 |
|  | ACAAAAATAACCAACCACTCACC |  |  |
| MR7-2 | GGTTAATTATTTTTAGAGAAATGAG | 56.8 | 408 |
|  | CCCCCAAAAAAATATACCTCAAAAT |  |  |
| MR8-1 | TGTTTTGATTAGTTATTTTTATAGTGGAGA | 59.9 | 319 |
|  | AACCAAAAATCCAAAACTACAACAT |  |  |
| MR8-2 | GAAAATTTTGATATGTTGTAGTTTTGGA | 59.9 | 247 |
|  | ATCAAAAAAACAAAAACCACCTCC |  |  |
| MR8-3 | GAGGTGGTTTTTGTTTTTTTGATG | 59.9 | 238 |
|  | AACCCCTCACTAACCTTATCACAAC |  |  |
| MR8-4 | TGTGATAAGGTTAGTGAGGGGTTAT | 59.9 | 232 |
|  | TAAAATCAAAAAAACCCAATCCTC |  |  |
| MR8-5 | ATTGGGTTTTTTTGATTTTAGTGAA | 59.9 | 328 |
|  | AAATACCCAACAAATCTCAAACTAAA |  |  |

**Supplemental Materials and Methods.**

**Mutation analyses**

Genomic DNAs were extracted from individual clinical samples and cell lines using the DNAiso Reagent (TaKaRa) according to the manufacturer’s protocol. Next-generation sequencing for *WT1* (exons 7 and 9), *TET2* (exons 3, 5-9 and 11) and *TP53* (exons 5-8) and data analyses were performed by the YUANQI BIO Co., Ltd (Shanghai, China). Nonsense and frameshift variations were all regarded as true mutations. Given some nonsynonymous SNVs (P29R, I1762V, V218M, L1721W, and H1778R) in *TET2* have been documented as SNPs in dbSNP (<http://www.ncbi.nlm.nih.gov/snp/)> and were not considered as true missense mutations in other papers, they were excluded from the subsequent analyses of this study.

**Cell lines and culture conditions**

Five AML cell lines (TF-1, U937, NB4, KG-1 and Kasumi-1) were purchased from the Institute of Biochemistry and Cell Biology of the Chinese Academy of Sciences (Shanghai, China). HL-60 and K562 were purchased from ATCC, and MOLM-13 was from AddexBio. TF-1, U937 and NB4 cell lines were cultured in RPMI 1640 medium supplemented with 10% fetal bovine serum (10% FBS), K562 cells were cultured in IMDM medium supplemented with 10% fetal bovine serum (10% FBS). Kasumi-1 and MOLM-13 cells were cultured in RPMI 1640 medium supplemented with 20% fetal bovine serum (20% FBS), HL-60 and KG-1 cell lines were cultured in IMDM medium supplemented with 20% fetal bovine serum (20% FBS). All cells supplemented with 100 U/ml penicillin and 100 mg/ml streptomycin (Invitrogen, Shanghai, China) in humidified air at 37°C with 5% CO_2_.

**Plasmids**

Plasmid for *MEG3* expression was purchase from addgene and then subcloned into pCDH-puro vector. Plasmid for PGL4-p53 was purchased from Promega. Plasmid for shRNAs against *TET2* were purchased from Sigma-Aldrich. DNA fragments of *TET2* catalytic domain (TET2^CD^) and *TET2* catalytic inactive mutant (TET2^CM^) were gifts from Dan Ye (Molecular and Cell Biology Laboratory, Institutes of Biomedical Sciences, Shanghai Medical College, Fudan University, China). Plasmids for *WT1* expression were gifts from Haixin Lei (Institute of Cancer Stem Cell, Cancer Center, Dalian Medical University, China). All expression constructs were verified by DNA sequencing.

**Lentiviral Transduction**

All plasmid vectors (pCDH-MEG3 and control vector) for transfection were extracted by Endo-free Plasmid Maxi Kit (OMEGA, USA). For lentivirus production, the pCDH-MEG3 and control vectors were cotransfected with packaging (psPAX2) and envelope (pMD2.G) vectors into HEK293T cells. Lentivirus was harvested from the supernatant at 36 hours post transfection, and mixed with 8 μg/ml polybrene to increase the infection efficiency. U937, HL-60 and MOLM-13 cell lines were infected with the lentivirus and selected in 2 μg/ml puromycin for 2 weeks.

**Transplantation of human leukemic cells**Female NOD-SCID mice (6-8weeks old) were randomly separated into different groups, and were injected with PBS, U937-CTRL or U937-MEG3 cells. All cells were washed twice in PBS, cleared of aggregates and debris using a 0.22 μm cell filter, and suspended in PBS at a concentration of 4 × 10^6^ cells per 200 μl of PBS per mouse. Daily monitoring of mice for symptoms of disease (paralysis, hunched back, weakness, reduced motility) determined the time of killing for injected animals with signs of distress. If no signs of distress were seen, mice were analyzed 8 weeks after injection except as otherwise noted.

**Assessment of leukemic engraftment**NOD-SCID mice were humanely killed in accordance with IACUC protocols. Bone marrow (mixed from tibias and femurs) were dissected in a sterile environment, flushed in PBS and made into single cell suspensions for analysis by flow cytometry (Accuri C6), and extract RNA and make bone marrow smear. Peripheral blood was obtained through retroorbital bleeds before killing to extract RNA and make peripheral blood smear. The bone marrow cells of mice were stained with human CD45 and CD38 specific antibodies. The detailed information of antibodies is summarized in Supplementary Table 3.

**Tumor formation assay in a nude mouse model**

Female athymic BALB/c nude mice aged 4 weeks were maintained under specific pathogen-free conditions. U937 cells were transfected with pCDH-MEG3 and control vector, washed with PBS, and resuspended at a concentration of 2 × 10^7^ cells/ml. A volume of 0.1 ml of suspended cells was subcutaneously injected into a single side of the posterior flank of each mouse. Tumor growth was examined every three days, and tumor volumes calculated using the equation V = 0.5 × D×d^2^ (V, volume; D, longitudinal diameter; d, latitudinal diameter). At 3 weeks post-injection, mice were euthanized, and the subcutaneous growth of each tumor examined.

All animals’ study was carried out in accordance with the National Institute of Health Guide for the Care and Use of Laboratory Animals under the approval of the SPF Laboratory Animal Center at Dalian Medical University. The protocol was approved by the Animal Care and Ethics Committee of Dalian Medical University. All surgery was performed under sodium pentobarbital anesthesia, and all efforts are made to minimize suffering in mice.

**RNA extraction and RT-qPCR analyses**

The total RNA was extracted from tissues or cultured cells with TRIzol reagent (Invitrogen), according to the manufacturer’s protocol. One microgram total RNA was reverse transcribed in a final volume of 20 μl using random primers under standard conditions using PrimeScript RT Reagent Kit with gDNA Eraser (TaKaRa). After the RT reaction, 2 μl of the complementary DNA was used for subsequent RT-qPCR reactions (SYBR Premix Ex Taq, TaKaRa) according to the manufacturer’s instructions. The results were normalized to the expression of *ACTB*. The RT-qPCR and data collection were carried out on Agilent Mx3005P real-time PCR system (Agilent Technologies). The primer sequences are listed in Supplementary Table 4.

**Cell proliferation assay**

Cell proliferation was monitored using MTT (Sigma-Aldrich). pCDH-MEG3/ siRNA-1 and control vector transfected AML cell lines (3000/well) were allowed to grow in 96-well plates. Cell proliferation was measured every 24 hours following the manufacturer’s protocol. All experiments were performed in quadruplicate.

**Flow-cytometric analysis of apoptosis**

AML cell lines transfected with pCDH-MEG3/siRNA-1 and control vector were harvested 48 hours after transfection. Following double staining with FITC-Annexin V and Propidium iodide (PI), the cells were analyzed using flow cytometry (FACScan®; BD Biosciences) equipped with a CellQuest software (BD Biosciences). Cells were discriminated into viable cells, dead cells, early apoptotic cells, and apoptotic cells. The percentage of apoptotic cells was compared to control groups from each experiment. All of the samples were assayed in triplicates.

**Immunoprecipitation and western blotting**

Cells were lysed in ice-cold NP-40 buffer [50 mM Tris-HCl (pH 7.4), 150 mM NaCl, 0.1% NP-40]] containing protease inhibitor cocktail (Millipore). Immunoprecipitation was carried out by incubating appropriate antibody with cell lysate for 1 hr., followed by incubating with Protein-G beads (Life Technologies) for another 2 hrs. at 4°C before beads were washed for three times with ice-cold NP-40 buffer. Standard western blot protocols were adopted. The detailed information of antibodies is summarized in Supplementary Table 3.

**Luciferase-reporter assay**

The luciferase assays were performed using a luciferase assay kit (Promega) according to the manufacturer’s protocol. Briefly, cells were first transfected with appropriate plasmids in 12-well plates. Next, the cells were collected and lysed for luciferase assay 48 h after transfection. The relative luciferase activity was normalized with renilla luciferase activity. The promoter region of MEG3 was PCR-amplified by TaKaRa LA Taq (TaKaRa) with the primers MEG3-p-F (Xho1 site) and MEG3-p-R (HindIII site), and was subcloned into the pGL4 basic firefly luciferase reporter. The amplified PCR fragments were then used as a template for generating promoter constructs. All PCR products were verified by DNA sequencing. The primer sequences are listed in Supplementary Table 4.

**Chromatin immunoprecipitation assay**

# The ChIP assays were performed using EZ-ChIP KIT according to the manufacturer^’^s instruction (Millipore). Briefly, cells were cross-linked with 1% paraformaldehyde and sonicated. Solubilized chromatin was immunoprecipitated with antibodies against WT1, Flag, or negative control IgG. Antibody-chromatin complexes were pulled down using Dynabeads® Protein G (Life Technologies) for Immunoprecipitation, washed and then eluted. After cross-link reversal and proteinase K treatment, immunoprecipitated DNA was extracted with phenol-chloroform, ethanol precipitated. The DNA fragments were further analyzed by RT-qPCR. ChIP primers are listed in Supplementary Table 4.

**Sodium bisulfite treatment and sequencing**

1.5 microgram of genomic DNA was treated with sodium bisulfite using the DNA Bisulfite Conversion Kit (TIANGEN) according to the manufacturer’s protocol. Eight cytosine-phosphate-guanosine (CpG)-rich regions were analyzed: two corresponding to the putative intergenic differentially methylated regions (MR1 and MR2), six upstream or within MEG3 (MR3-8). PCRs were used under the following conditions: 94 ℃ for 2 min, 94℃ for 30 sec, 64.5℃ (see Supplementary Table 5 for Tms) for 30 sec, and 68℃ for 1 min for 40 cycles, and 68℃ for 10 min. PCR products were subcloned into a pGEM®-T Easy Vector System (Promega), and constructs representing each region from each sample were randomly selected for sequence analysis.
